# Recent Advances in Stimulus‐Responsive Nanocarriers for Gene Therapy

**DOI:** 10.1002/advs.202100540

**Published:** 2021-05-16

**Authors:** Cheng Yu, Long Li, Pei Hu, Yan Yang, Wei Wei, Xin Deng, Lu Wang, Franklin R. Tay, Jingzhi Ma

**Affiliations:** ^1^ Department of Stomatology Tongji Hospital Tongji Medical College Huazhong University of Science and Technology Wuhan Hubei Province 430030 China; ^2^ Department of Oncology Tongji Hospital Tongji Medical College Huazhong University of Science and Technology Wuhan Hubei Province 430030 China; ^3^ The Graduate School Augusta University Augusta GA 30912 USA

**Keywords:** gene therapy, nonviral vector, stimulus‐responsive nanocarriers, viral vector

## Abstract

Gene therapy provides a promising strategy for curing monogenetic disorders and complex diseases. However, there are challenges associated with the use of viral delivery vectors. The advent of nanomedicine represents a quantum leap in the application of gene therapy. Recent advances in stimulus‐responsive nonviral nanocarriers indicate that they are efficient delivery systems for loading and unloading of therapeutic nucleic acids. Some nanocarriers are responsive to cues derived from the internal environment, such as changes in pH, redox potential, enzyme activity, reactive oxygen species, adenosine triphosphate, and hypoxia. Others are responsive to external stimulations, including temperature gradients, light irradiation, ultrasonic energy, and magnetic field. Multiple stimuli‐responsive strategies have also been investigated recently for experimental gene therapy.

## Graphical abstract

Genetic diseases and cancer bring grief to those who suffer from these ailments. Nonviral nanocarriers decorated with stimuli‐responsive elements are now used in gene therapy to deliver good genes via the blood circulation to destined sites where the cargoes are released upon stimulation to replace the bad genes and alter cell functions.

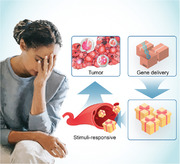

## 1 Introduction

Many human diseases are linked to genetic changes.^[^ ^1^ ^]^ Gene therapy represents a breakthrough in the management of these genetically altered illnesses that paths the future direction for the treatment of ailments that cannot be cured by conventional medicine. As of December, 2020, many gene therapy drugs have been approved globally (**Table** **1**).^[^ ^2^, ^3^ ^]^ Cellular and gene therapy products approved by the United States Food and Drug Administration (US FDA) include Kymriah and Yescarta for the treatment of acute lymphoblastic leukemia, Luxturna for the treatment of Leber congential amaurosis, Patisiran for the treatment of polyneuropathy in people with hereditary transthyretin‐mediated amyloidosis, and Zolgensma for the treatment of spinal muscular atrophy (https://www.fda.gov/vaccines‐blood‐biologics/cellular‐gene‐therapy‐products/approved‐cellular‐and‐gene‐therapy‐products; accessed 3‐6‐2021).^[^ ^4^, ^5^ ^]^ With the development of gene therapy for children with Duchenne malnutrition^[^ ^6^ ^]^ and eye diseases,^[^ ^7^ ^]^ it is surmised that gene therapy will soon be universally adopted clinically. Because nucleic acids are easily degraded by enzymes in vivo, safe and effective vectors are required to deliver them to target cells.^[^ ^8^ ^]^ Current commercial delivery vectors for nucleic acids are predominantly viral vectors, with the most common vector being adeno‐associated virus (AAV). Virus vectors are not only expensive, their security has long been a common concern. Currently, the world's most costly gene therapy drug is Zolgensma, commercially available from Novartis at more than US$2.1 million per dose.^[^ ^9^ ^]^ The risk of insertional mutagenesis by viral vectors is dose‐dependent and may lead to serious side effects.^[^ ^10^ ^]^ Between May and August, 2020, deaths caused by sepsis were reported in three children who were administered in a clinical trial with high doses of AT132 (Resamirgene bilparvovec) delivered via adenovirus vectors for treatment of X‐linked myotubular myopathy (Statements from Audentes; https://www.joshuafrase.org/get‐involved/recensus‐study.php; accessed 11‐28‐2021).

**Table 1:** Examples of approved gene therapy products worldwide

Company/institute^a)^	Product	Approved country	Year	Vector employed	Indication/disease
Shenzhen SiBiono Gene Tech	Gendicine	China	2003	Adeno‐associated virus	Head and neck cancer
Shanghai Sunway Biotech	Oncorine	China	2005	Oncolytic virus	Head and neck and esophagus cancer, nasopharyngeal cancer
Epeius Biotechnologies	Rexin‐G	USA	2010	Retrovirus	Metastatic solid tumors
Human Stem Cells Institute	Neovasculgen	Russia	2012	Cytomegalovirus	Atherosclerotic peripheral arterial disease
Amgen	Imlygic	USA	2015	HSV 1	Melanoma
GlaxoSmithKline	Strimvelis	EU	2016	Retrovirus	Adenosine deaminase deficiency; severe combined immune deficiency
MolMed SPAA	Zalmoxis	EU	2016	Retrovirus	Hematopoietic stem cell transplantation graft vs host disease
Novartis	Kymriah	Switzerland/USA	2017	Lentivirus	B‐cell acute lymphoblastic leukemia
Gilead/Kite Pharma	Yescarta	USA	2017	Retrovirus	Diffuse large B‐cell lymphoma
Roche/Spark Therapeutics	Luxturna	USA	2017	Adeno‐associated virus	Biallelic RPE65 mutation‐associated retinal dystrophy
KolonTissueGene	Invoss	South Korea	2017	Retrovirus	Knee osteoarthritis
Alnylam Pharmaceuticals Inc.	Patisiran	USA/EU	2018	Liposome	Familial amyloid polyneuropathy
Novartis/AveXis	Zolgensma	Switzerland/USA	2019	Adeno‐associated virus	Spinal muscular atrophy
Bluebird Bio	Zynteglo	EU	2019	Lentivirus	Transfusion‐dependent *β*‐thalassemia
SSM Cardinal Glennon Children's Medical Center	ALLOCORD	USA	2019	NA	Hematopoietic disorders
Vericel Corporation	Matrix‐induced autologous chondrocyte implantation (MACI)	USA	2019	NA	Single/multiple symptomatic, full‐thickness cartilage defects of the knee with or without bone involvement
Dendreon Corporation	Provenge	USA	2019	NA	Asymptomatic or minimally symptomatic metastatic castrate resistant (hormone refractory) prostate cancer
Kite Pharma, Inc.	Tecartus	USA	2020	Retrovirus	Relapsed/refractory mantle cell lymphoma.

^a)^ Antisense therapy using direct injection of antisense oligonucleotides and whole cell therapy are not included in the table. Abbreviations: EU, European Union; HSV, Herpes simplex virus Type 1; Inc., Incorporated; Tech, Technology; USA, United States of America.

Many strategies have been proposed and developed for gene delivery.^[^ ^11^ ^]^ Among these strategies, nonviral vectors fare better in controlling costs and adverse reactions. Stimulus‐responsive nanoparticle delivery systems are one of the most extensively studied nonviral vectors. They participate in transfection of nucleic acids via responding to different forms of internal and environmental stimuli. While there are excellent recent reviews on polymeric stimulus‐responsive systems, they mostly address the use of polymeric systems^[^ ^12^, ^13^ ^]^ for drug delivery and tumor imaging.^[^ ^14^ ^]^ With the advent of gene therapy, we see the need for a review that focuses specifically on the development and use of stimulus‐responsive nanocarriers for gene delivery over the last 5 years.

## 2 Gene Therapy

### 2.1 Basic Concepts, History, and Ongoing Clinical Trials

**Figure 1:**
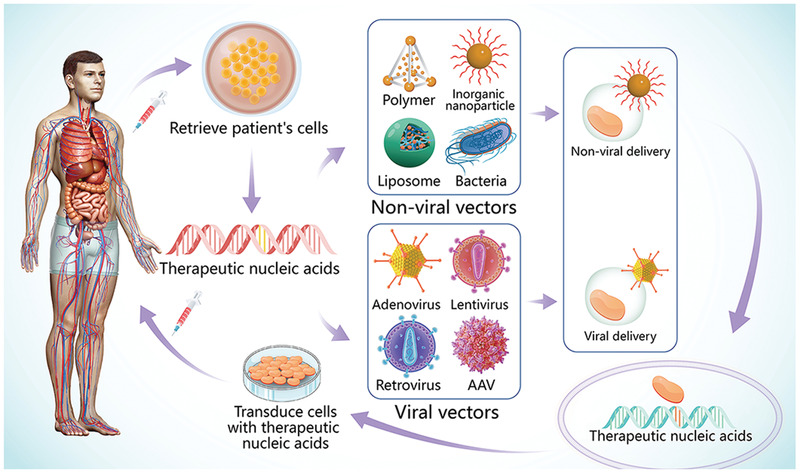
Overview of gene therapy strategies. Cells are retrieved form the patient. Therapeutic nucleic acids are introduced into the retrieved cells via viral or nonviral vectors. The modified cells are then re‐administered genetically and amplified, and subsequently re‐infused into the patient. Finally, therapeutic nucleic acids are delivered directly to the patient's target cells by viral or nonviral vectors. Abbreviation: AAV, adeno‐associated virus.

The goal of gene therapy is to enable expression of therapeutic nucleic acids [deoxyribonucleic acid (DNA), ribonucleic acid (RNA), or oligonucleotides] in the target cells of a patient, to relieve symptoms caused by defective gene expression.^[^ ^11^ ^]^ To date, inducing the expression of a certain gene or protein is realized through the introduction of plasmid DNA (pDNA) or messenger RNA (mRNA) into target cells, while the suppression a certain gene is realized through small interfering RNA (siRNA) or RNA interference (RNAi) technology. Two basic strategies have evolved: in vitro and in vivo gene delivery (**Figure** **1**). For ex vivo delivery strategies, therapeutic nucleic acids are introduced into cells, amplified in vitro and subsequently re‐infused into the patient. For in vivo delivery strategies, therapeutic nucleic acids are encapsulated in specific vectors and delivered directly to the patient's target cells.^[^ ^15^ ^]^

**Figure 2:**
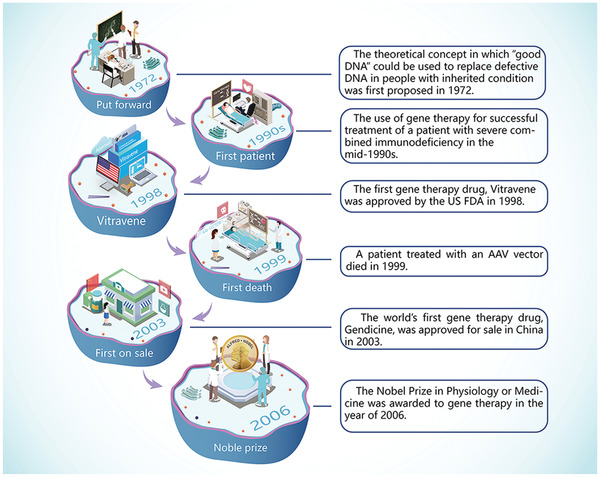
Milestones in the development of gene therapy. The theoretical concept in which “good DNA” can be used to replace defective DNA in human subjects with inherited conditions was first proposed in 1972. The use of gene therapy for successful treatment of a patient with severe combined immunodeficiency was first contemplated in the mid‐1990s. The first gene therapy drug, Vitravene, was approved by the US FDA in 1998. A patient treated with an AAV vector died in 1999. The world's first gene therapy drug, Gendicine, was approved for sale in China in 2003. The Nobel Prize in Physiology or Medicine was awarded to gene therapy in 2006. Abbreviation: AAV, adeno‐associated virus.

**Figure** **2** represents an abridged timeline for the development of gene therapy. The theoretical concept in which “good DNA” could be used to replace defective DNA in people with inherited condition was first proposed by Drs. Theodore Friedmann and Richard Roblin in 1972.^[^ ^16^ ^]^ Fueled by its appealingly straightforward approach, the concept of gene therapy soon captured scientists’ interest and the public's imagination. Blaese and colleagues reported the use of gene therapy for successful treatment of a patient with severe combined immunodeficiency in the mid‐1990s.^[^ ^17^ ^]^ The major crises associated with gene therapy have all been historically associated with the use of gene carriers. Development of gene therapy was hampered by the death of a patient treated with an AAV vector in 1999.^[^ ^18^ ^]^ Vitravene, the first gene therapy drug approved by the US FDA in 1998, was used for topical treatment of retinitis in patients with immune dysfunction.^[^ ^19^ ^]^ Although Vitravene was subsequently withdrawn from the market, interests in gene therapy rekindled with the advent of new RNA gene therapy technology and the introduction of liposome vectors.^[^ ^20^ ^]^ The world's first gene therapy drug, a recombinant human p53 oncolytic adenovirus named Gendicine, was designed for the treatment of head and neck tumors. Gendicine was approved for sale in China in 2003.^[^ ^2^ ^]^ Hopes in the future of gene therapy were boosted when the Nobel Prize in Physiology or Medicine for 2006 was awarded jointly to Andrew Fire and Craig Mello for their groundbreaking discovery that double‐stranded RNA can selectively silent genes, the basic tenet for RNAi technology.^[^ ^21^ ^]^ Glybera was approved as a commercially available gene therapy product for lipoprotein lipase deficiency by the European Medicines Agency in 2012.^[^ ^22^ ^]^ Glybera, which was also delivered using an AAV vector, was withdrawn in 2017 because of its high price and rare indications.^[^ ^23^ ^]^ Chimeric antigen receptor‐T cell (CAR‐T) therapy, an in vitro gene delivery strategy, has been reported to be effective in the treatment of adults with relapsed/refractory large B cell lymphoma and pediatric and young adult patients with B cell precursor acute lymphoblastic leukemia that is refractory or in second/later relapse.^[^ ^24^ ^]^ The CAR‐T cells are engineered from a patient's own T cells to identify target antigens and trigger an immune response. The world's first RNAi drug Patisiran, consisting of a 19‐base‐pair, double‐stranded siRNA coupled with lipid nanoparticles, was approved by the US FDA in 2018,^[^ ^4^, ^25^ ^]^ while Zolgensma approved in 2019. In addition to the US FDA, more than ten gene therapy drugs have been approved worldwide.^[^ ^26^ ^]^ However, the clustered regularly interspaced short palindromic repeats (CRISPR)‐baby scandal in China in 2018 (twin girls born with CRISPR‐edited genomes in which a gene known as *CCR5* was disabled to prevent entry of the human immunodeficiency virus into cells) was severely criticized by the scientific community as well as the lay public. Such an unfortunate episode suggests that gene therapy is a double‐edged sword and needs to be more strictly regulated.^[^ ^27^ ^]^

**Figure 3:**
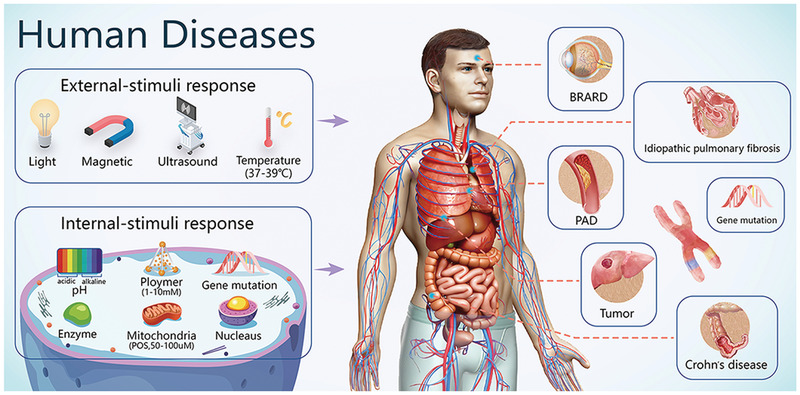
The use of gene therapy for treating human diseases. Approved gene therapies and clinical trials have been conducted for human diseases that include multiple solid tumors, single‐gene diseases (e.g., BRARD), cardiovascular diseases (e.g., PAD), and idiopathic diseases (e.g., idiopathic pulmonary fibrosis and Crohn's disease). Abbreviations: BRARD, Biallelic RPE65‐associated retinal dystrophy; PAD, atherosclerotic peripheral arterial disease.

As of November 2020, a search on ClinicalTrials.gov (https://www.clinicaltrials.gov/) identified nearly 4500 clinical trials that had been approved for gene therapy. Of these clinical trials, 1645 had been completed and 545 were in phase 3/4. This indicates that gene therapy technology is developing rapidly and is gradually translating into clinical practice. Most of the phase 3/4 clinical trials focused on tumors, single‐gene diseases, cardiovascular diseases, and infectious diseases (**Figure** **3**). In these clinical trials, the lack of a safe and efficient delivery vector remains the most critical bottleneck that restricts the development of gene therapy technology. Although viral vectors remain by far the most popular delivery strategy, nonviral vectors are becoming more common.^[^ ^28^, ^29^, ^30^, ^31^ ^]^

### 2.2 Delivery Systems

The half‐lives of naked nucleic acids are very short because they are rapidly degraded by chemical substances during circulation.^[^ ^32^ ^]^ In addition, negatively charged macromolecules such as DNA are difficult to get close to negatively charged cell membranes.^[^ ^33^ ^]^ This results in low uptake of the delivered nucleic acids by target cells. Protein/nucleic acid complexes such as Cas9 (positively charged)‐synthetic single‐guide RNA (sgRNA; negatively charged) are much more challenging to deliver than DNA or mRNA because of their mixed charges.^[^ ^34^ ^]^ Accordingly, well‐designed systems are essential for effective delivery of these genetic materials for transfection or genome editing. Regrettably, fail‐safe gene delivery systems are still currently lacking for these materials.

**Figure 4:**
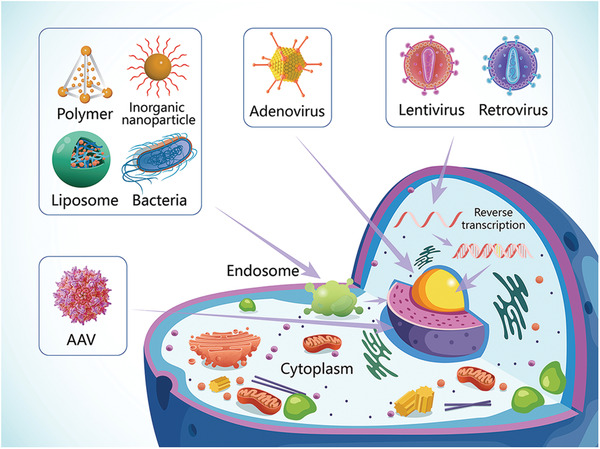
Major types of gene therapeutics and delivery strategies. Vector‐based delivery systems include virus and nonvirus vectors. Virus vectors include lentivirus, adenovirus, and adeno‐associated virus (AAV). Adenovirus is capable of transducing its DNA by endocytosis into nucleus of the host. Both lentivirus and retrovirus belong to the Retrovirus family. Their RNA can be transcribed into complementary DNA (cDNA) and the copies of cDNA copy can be readily integrated into the host cell genome. Adeno‐associated virus can be efficiently integrated into human chromosome with helper virus. Other nonviral based delivery vectors, including bacteria and nanoconstructs (such as inorganic nanoparticles, polymers, and liposomes) loaded with nucleic acids can enter tumor cells via endocytosis to release therapeutics.

Gene delivery systems may be divided into physically mediated and vector‐based systems. Physical delivery systems such as gene guns and microneedles are relatively simple in design, but require chemical modification of the nucleic acids to prevent their degradation.^[^ ^35^, ^36^ ^]^ Vector‐based delivery systems include virus and nonvirus vectors (**Figure** **4**). The ideal vector should meet two requirements simultaneously. The first requirement is to protect the nucleic acid from degradation and provide prolonged performance in circulation. The second requirement is to promote the recognition of target cells and improve cellular uptake and intracellular escape.^[^ ^37^ ^]^

#### 2.2.1 Viral Vector Delivery

To date, all gene therapy drugs that are approved for clinical use, with the exception of Patisiran, utilize a viral delivery system (Table 1). Adeno‐associated virus and retrovirus vectors are extensively used because of their high transfection efficacy. In 1995, an AAV vector was used for the first time in the treatment of cystic fibrosis in human patients. After years of continuous research and improvement, AAV vectors have emerged as the leading platform for gene delivery in the treatment of a variety of human diseases.^[^ ^38^ ^]^ However, 20–80% of individuals reported having antibodies to AAVs in their blood, which, even at low titers, prevent the AAVs from entering target cells.^[^ ^39^ ^]^ Lentivirus vectors are the most widely used retrovirus vectors. Third generation self‐inactivated lentivirus vectors have recently been used in different clinical trials. They play an important role in the correction of primary immunodeficiency and hemoglobin‐related diseases, as well as in the modification of chimeric antigen receptors.^[^ ^40^ ^]^ The shortcomings of viral vectors are their limited capacity to carry genes (particularly those with a large number of base pairs; see Section 5) and potential safety issues.^[^ ^41^ ^]^

#### 2.2.2 Nonviral Vector Delivery

Compared with viral vectors, nonviral vectors have the advantages of low cost, simple preparation, facile mass production, safety and the potential capability to delivery an unlimited length of exogenous genes. In addition, they are easier to store and have longer shell‐lives because of their stability.^[^ ^42^, ^43^ ^]^ Nanoparticles are materials with a size ranging from 10 to 1000 nm. Their enhanced permeability and retention render them highly sought after in research on nonviral vector delivery.^[^ ^44^, ^45^ ^]^ Nanoparticles also have higher surface area‐to‐volume ratios, better biocompatibility, relatively acceptable stability, controlled surface charge, and longer blood circulation times.^[^ ^46^, ^47^ ^]^ These favorable properties render the promising gene delivery vehicles.

Nonviral vectors may be further divided into organic and inorganic materials. Examples of organic materials include liposomes, polyethyleneimine (PEI) and its derivatives, cationic polypeptides, dendrimers and their derivatives, chitosan, and its derivatives, polyurethane, as well as cyclodextrin and its derivatives. Examples of inorganic materials include gold nanoparticles, carbon nanotubes, graphene, quantum dots, upconverted nanoparticles, and silica nanoparticles.^[^ ^48^ ^]^ Liposomes are phospholipid bilayer vesicles containing two different environmental niches (hydrophobic and hydrophilic). Hence, they have the capability to deliver both hydrophobic and hydrophilic molecules.^[^ ^49^ ^]^ Liposomes are used extensively for the delivery of RNA and DNA. Cationic liposomes, the most common nonviral gene delivery material, have been commercialized as in vitro gene transfection agents for nearly two decades.^[^ ^50^ ^]^ Although they are more toxic than neutral liposomes, the have higher gene loading capacity and transfection capability. In addition, the toxicity of cationic liposomes may be improved by surface modification with biocompatible polymers such as poly(ethylene glycol) (PEG).^[^ ^51^ ^]^ Cationic lipids are used for loading Cas9‐sgRNA complexes in vivo because it is difficult to be loaded by other common carriers.^[^ ^52^ ^]^ Another common category of nanocarriers are polymers. Polymers are attractive because of their diversity in structure, composition and functional groups.^[^ ^12^ ^]^ Cationic polymers bind negatively charged nucleic acids electrostatically to form DNA/RNA–polymer complexes.^[^ ^53^ ^]^ Many cationic polymers such as polylysine,^[^ ^54^ ^]^ poly(amino esters),^[^ ^55^ ^]^ and polyamine dendrimers^[^ ^56^ ^]^ have been used as nanocarriers for experimental gene therapy. Mesoporous silica nanoparticles are inorganic nanoparticles with large surface area, pore volume, and biodegradability. They have attracted much attention in nanomedicine over the last 5 years.^[^ ^57^ ^]^

Although progress has been achieved in the development of nonviral vectors for gene transfection, these systems are still far less efficient than virus‐based systems. Because of the complexity of delivery pathways and cell barriers, the precise structural requirements of effective gene delivery vectors remain a challenge.

## 3 Biological Barriers for Nanocarriers in Gene Therapy

During the process of gene delivery, gene vectors encounter different physiological barriers such as blood circulation, opsonization, and endocytosis by the mononuclear phagocytic system, tissue pressure, endosomal escape, and intracellular transmission difficulties.^[^ ^58^, ^59^ ^]^ These barriers may capture and/or destroy the therapeutic nucleic acids cargo before it reaches the target. Strategies have been instigated to tackle these problems through years of nanomaterials research. For example, surface charge shielding is achieved with acid or enzyme‐separable PEGylation to enhance steric stability.^[^ ^60^ ^]^ PEGylation refers to the process of covalent/noncovalent attachment of PEG polymer chains to molecules and macrostructures. Although PEGylated nanocarriers are capable of prolonging circulation and reducing side effects, they still have deficiencies known as the PEG dilemma.^[^ ^61^ ^]^ Problems include low cellular uptake and incomplete release of genes to target cells. Furthermore, repeated injections of PEGylated lipids in the same animal induce the phenomenon of accelerated blood clearance. Although cellular uptake may be enhanced by attaching targeted ligands to complexes, and release may be improved by constructing acid labile bonds between neutral shields and cation complex nuclei, solutions to these problems require robust refinement before they can be brought to clinical testing.^[^ ^62^ ^]^ The pores of tumor microvessels are reported to be between 200 and 2000 nm in diameter.^[^ ^41^ ^]^ The distribution of nanoparticles after systemic administration is significantly correlated with their size. Nanoparticles over 200 nm tend to accumulate in the liver and spleen.^[^ ^63^, ^64^ ^]^ Improvements can be made to the size of the nanocarriers. Vascular endothelial cells are sparsely arranged within the tumor environment, where the internal lymphatic drainage is insufficient and the blood flow rate is low. Once the nanoparticles enter the vessels inside a tumor, they will be retained in the tumor site. This phenomenon is known as the “enhanced permeability and retention effect,” which is generally perceived as the major reason for the accumulation of nanoparticles in a tumor site.^[^ ^65^ ^]^ This theory, however, was challenged this year with supportive data suggesting that active endocytosis by endothelial cells may be the key mechanism for nanoparticle accumulation at the tumor sites.^[^ ^66^ ^]^ This novel finding may provide new ideas for the design of nanocarriers.

**Figure 5:**
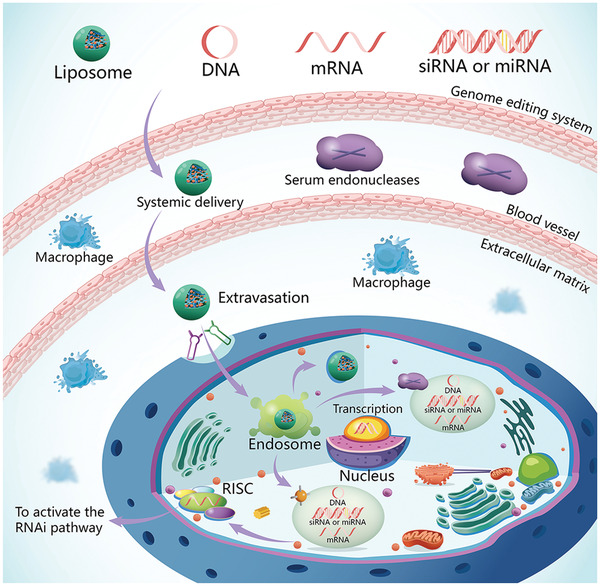
Barriers to successful in vivo delivery of nucleic acids. After they are uptaken into cells by endocytosis, the delivered nucleic acids are liable to be degraded by the enzyme‐rich, acidic environment of the intracellular endosomes and lysosomes. Incorporation of modified PEI in nanoparticles helps to release complex nucleic acids into the cytoplasm to improve transfection efficacy. Abbreviations: RISC, RNA‐induced silencing complex; PEI, polyethylenimine.

An important obstacle to intracellular delivery is that the delivered nucleic acids are liable to be degraded by the enzyme‐rich, acidic environment of the intracellular endosomes and lysosomes, after they are uptaken into cells via endocytosis (**Figure** **5**). Polyethylenimine exerts a unique proton sponge effect to promote endosomal escape. Hence, incorporation of PEI in nanoparticles helps to release complex nucleic acids into the cytoplasm to improve transfection efficacy. Nevertheless, the cytotoxicity of PEI limits its in vivo application.^[^ ^67^ ^]^ Because cell membranes are damaged by the positively charged PEI, changing the surface properties of PEI through neutralization or hybridization with other biocompatible polymers, such as shielding of its positive surface charges, can reduce its toxicity and enhance in vivo stability.^[^ ^68^ ^]^ The nonbiodegradable nature of PEI is another important limitation of this molecule.^[^ ^69^ ^]^ Crosslinking of PEI with stimulus‐responsive material can solve this problem to some extent.^[^ ^68^, ^70^ ^]^ The modified PEI is often used as a core component in the design of more complex gene nanocarriers.^[^ ^71^ ^]^

Delivery of different types of genes relies on different carriers for delivery. Examples of carriers used to satisfy these different requirements are summarized in **Table** **2**.

**Table 2:** Delivery requirements for different types of genes. Abbreviations; AAV, adeno‐associated virus; CD19, cluster of differentiation 19; miR, microRNA; MMP‐9, matrix metalloproteinase‐9; NF‐*κ*B, nuclear factor kappa‐light‐chain‐enhancer of activated B cells; OPN, osteopontin; pCMFO, plasmid for chronic multifocal osteomyelitis; PDCD4, programmed cell death 4; PEI, polyethylenimine; P‐gp, P‐glycoprotein; TLR4, Toll‐like receptor 4; TRAIL, tumor necrosis factor‐related apoptosis‐inducing ligand; TTR, transthyretin; VEGF, vascular endothelial growth factor

Gene types	Delivery requirement	Treatment targets	Carriers	Application
siRNA	Protects siRNA from rapid degradation by nucleases and endosomes; helps siRNA penetrate negatively charged cell membranes, endothelium and tissues	TTR siRNA^[^ ^4^ ^]^	Liposome	Peripheral nerve diseases caused by hereditary amyloidosis of transthyretin
		VEGF SiRNA^[^ ^72^ ^]^	PEI	Lung cancer, liver cancer, breast cancer
		OPN siRNA^[^ ^73^ ^]^	Polymer	Breast cancer
		Survivin siRNA^[^ ^74^ ^]^	PEI	Prostate cancer
shRNA	Low toxicity, high drug loading and high delivery efficacy.	NF‐*κ*B shRNA^[^ ^75^ ^]^	PEI	Breast cancer
		MMP‐9 shRNA^[^ ^76^ ^]^	Polymer	Breast cancer
		shTLR4^[^ ^77^ ^]^	Liposome	Pancreatic fibrosis
miRNA	High delivery efficiency and stability	miR‐21^[^ ^78^ ^]^	Polymer	Glioblastoma
		P‐gp miRNA^[^ ^79^ ^]^	PEI	Breast cancer
mRNA	Have to be reverse transcribed into cDNA	Cyclin G1^[^ ^80^ ^]^	Retrovirus	Sarcoma
DNA	Improve transfection efficacy and therapeutic effects	TRAIL^[^ ^81^ ^]^	PEI	Liver cancer
		PDCD4^[^ ^82^ ^]^	Chitosan	Liver cancer
pDNA	High drug loading and high delivery efficacy	CD19^[^ ^83^ ^]^	Lentivirus	Acute lymphoblastic leukemia
		p53^[^ ^84^ ^]^	AAV	Head and neck squamous cell carcinoma
		pCMFO^[^ ^85^ ^]^	Liposome	*Mycobacterium tuberculosis* infection

## 4 Stimulus‐Responsive Nanocarrier Delivery Systems

A nanocarrier needs to overcome the aforementioned obstacles to reach the target cell nucleus or cytoplasm. One of the most effective ways to improve carrier release efficacy is to utilize extracellular environmental cues for signal stimulation.^[^ ^86^ ^]^ Compared with nonstimulus‐responsive nanocarriers, stimulus‐response delivery systems demonstrate more dynamic activities, which render vector release more precise. For example, a self‐assembled nanoparticle utilized pH‐sensitive amino lipids to deliver therapeutic ABCA4 pDNA as an in vivo gene therapy approach for treating Stargardt macular degeneration, a genetic eye disorder caused by mutations in the *ABCA4* gene that causes progressive vision loss.^[^ ^87^ ^]^

Stimuli used for triggering cargo release from gene carriers may be classified into two categories: 1) internal stimuli, which refer to naturally occurring changes in physiological and pathological conditions in cells or tissues (e.g., changes in temperature, redox potential, enzyme activity, or pH), and 2) external stimuli, which refer to externally induced changes in biological systems (e.g., electrical, magnetic, or ultrasonic stimulus).^[^ ^14^ ^]^ Because nucleic acids are easily degraded and premature release of nucleic acids may lead to the loss of therapeutic function at the cellular uptake stage, the design of stimulus‐response gene delivery is not exactly the same as stimulus‐response drug delivery. Many advances have been made on using stimulus‐responsive nanocarriers for gene therapy. Advances in the development of these vectors over the last 5 years are summarized below.

### 4.1 Internal Biologically Responsive Nanomaterials

#### 4.1.1 pH‐Responsive Nanocarriers

The acidity of tissues in pathological conditions, such as inflammation or tumors, is often lower than that of normal tissue and blood. There are also differences in pH between different intracellular substructures.^[^ ^88^ ^]^ In particular, endosomes and lysosomes have lower pH values that the rest of the cytoplasm. pH‐sensitive nanoparticles can deliver therapeutic nucleic acids to target tissues by taking advantage of this naturally diverse pH environment. Such a strategy has been investigated extensively in gene therapy research.^[^ ^89^ ^]^ pH‐based nanoscopical delivery systems have made significant breakthroughs in the diagnosis and treatment of infections and malignant tumors. These systems have also made many experimental advances in gene therapy. Different types of nanomaterials have been employed for designing pH‐responsive delivery systems, including polymer nanoparticles, liposomes, and inorganic nanoparticles. For example, lipid‐coated calcium phosphate nanoparticles successfully reduced the expression of programmed cell death protein‐1 (PD‐1) in tumor‐infiltrating lymphocytes.^[^ ^90^ ^]^

pH‐responsive nanoparticles are synthesized by the incorporation of ionizable pH‐sensitive functional groups (e.g., amines, carboxylic acids) or acid‐unstable chemical bonds (e.g., hydrazones, ester bonds) to respond at a specific pH value.^[^ ^91^, ^92^ ^]^ Nanocarriers modified with acid‐dependent insertion peptides, the conformations of which are altered in an acidic environment, represent another method for constructing pH‐responsive delivery systems.^[^ ^93^ ^]^ pH‐responsive nanocarriers have advantages in the delivery of nucleic acids such as pDNA and siRNA. These advantages include biocompatibility, biodegradability, stability in blood circulation, enhanced cellular uptake, and low cytotoxicity.^[^ ^94^, ^95^, ^96^, ^97^ ^]^

Polymers are common examples of pH‐response nanocarriers, which include chitosan,^[^ ^98^ ^]^ anion polymers such as poly(acrylic acid)^[^ ^99^ ^]^ and cationic polymers such as poly(l‐lysine).^[^ ^100^ ^]^ Chitosan is a natural product with many amine functional groups and positive charges. It possesses good biodegradability, biocompatibility, and its immunogenicity is lower than other polymers. Positively charged chitosan binds to negatively charged polymers, nucleic acids, and membranes. This makes it valuable as nanocarriers in pH‐sensitive gene delivery systems.^[^ ^101^ ^]^ A chitosan‐based gene carrier gel comprising silicon, PEI, and chitosan has been synthesized.^[^ ^102^ ^]^ Silicon is included in the delivery system to improve the hardness of the nanogel. Polyethylenimine is incorporated to enhance transfection efficacy because of its proton sponge effect.

The transfection efficacy of cationic liposomes is related to the properties of pH‐sensitive linkers such as carbamate, imidazole and ester.^[^ ^103^ ^]^ In vivo experiments showed that the pH‐sensitive cationic CL4H6‐lipid nanoparticles (CL4H6‐LNPs) were more effective in gene silencing than ONPATTRO (patisiran), a US FDA‐approved agent for the treatment of polyneuropathy associated with hereditary transthyretin‐mediated amyloidosis in adults. CL4H6‐LNPs have been optimized as nanocarriers for targeting hepatocytes.^[^ ^104^ ^]^ These nanoparticles also target macrophages. Apart from possessing antitumor therapeutic effect, CL4H6‐LNPs also reverse the protumor function of macrophages, with broad clinical advantages in tumor immunotherapy.^[^ ^105^ ^]^ The first lipid‐based siRNA drug approved by the US FDA, in which the siRNA is encapsulated in a cationic lipid coded‐named DLin‐MC3‐DMA ((6Z,9Z,28Z,31Z)‐heptatriacont‐6,9,28,31‐tetraene‐19‐yl 4‐(dimethylamino)butanoate), is sensitive to pH stimulation.^[^ ^106^ ^]^ Inspired by viral vectors, peptides can be actively delivered into cells to support efficient and safe gene delivery.^[^ ^107^ ^]^ The synergistic effect between the cell‐penetrating peptide octa‐arginine and the recently developed pH‐sensitive cationic lipid YSK05 makes it an efficient gene delivery system.^[^ ^108^ ^]^ Degradable inorganic calcium phosphate nanocarriers possess pH sensitivity and can rupture lysosomal membrane. This enables the nucleic acids to be released (i.e., escape) from the lysosomes.^[^ ^109^ ^]^

Tumor chemotherapy often produces drug resistance. Apart from inhibiting oncogenes, siRNA technology also targets genes related to tumor drug resistance and functions synergistically with chemotherapy. The co‐delivery of genes with chemotherapeutics represents a new, promising antitumor strategy.^[^ ^110^ ^]^ Such a platform is provided by nonviral vectors and many pH‐sensitive candidates have become available in recent years. The pH‐sensitive nonviral vector, carboxymethyl chitosan‐modified liposome, is capable of simultaneously loading sorafenib, a chemotherapeutic drug for treating kidney, liver and thyroid cancer, and Cy3 dye‐labeled siRNA. This enhances downregulation of vascular endothelial growth factor and induces early cell apoptosis.^[^ ^111^ ^]^ Polymers are also potential candidates for co‐delivery of genes and chemotherapeutics. A pH‐sensitive amphiphilic polymer micelle, poly[(1,4‐butanediol)‐diacrylate‐*β*‐*N*,*N*‐diisopropylethylenediamine]‐PEI, is stable in neutral pH and tumor extracellular pH. This amphiphilic polymer releases Taxol (paclitaxel) and protein kinase B (Akt) siRNA into the acid environment within lysosomes. The combination is capable of inhibiting the expression of genes that express protein kinase B and P‐glycoprotein 1, as well as inducing apoptosis of breast cancer cells.^[^ ^112^ ^]^ In addition to synergistic antitumor effects, co‐delivery has been used for management of nontumorous diseases. For example, a pH‐sensitive copolymer synthesized from PEG and PEI is used to co‐deliver simvastatin and siRNA to promote osteogenesis.^[^ ^98^ ^]^

**Figure 6:**
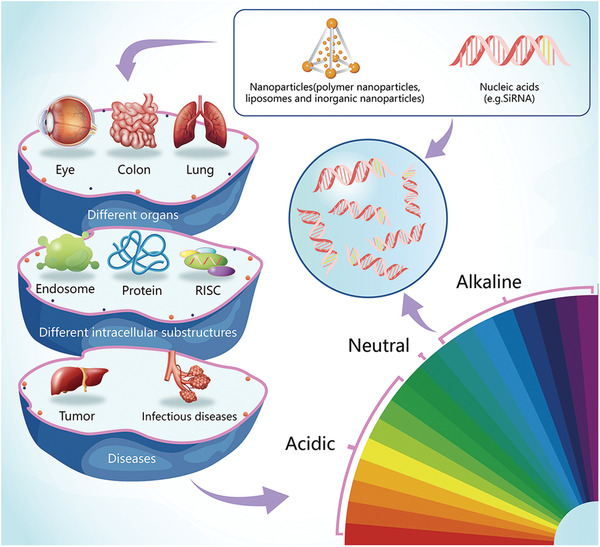
pH‐responsive nanocarriers for gene therapy. There are differences in pH between different organs, different intracellular substructures, and pathological conditions. Intracellular substructures, such as endosomes and lysosomes, have lower pH values than the rest of the cytoplasm. The acidity of tissues in pathological conditions such as inflammation or tumors is often lower than that of normal tissue and blood.

pH‐sensitive nanocarriers are used to prevent premature degradation of nucleic acids in intracellular organelles such as endosomes and lysosomes (**Figure** **6**). These vectors respond to the reduced pH by breaking down the membrane of the endosome, enabling gene release into the cytoplasm. However, there is still a lack of research on variation in pH among different tumors. Further efforts are urgently required to expedite the development of this class of nanocarriers for clinical application.

#### 4.1.2 Redox‐Responsive Nanocarriers

There are many reducing substances in the intracellular environment, such as the nicotinamide adenine dinucleotide phosphate system, the glutathione system and the oxygen/superoxide system.^[^ ^14^ ^]^ Glutathione is the main reducing substance in cells. The concentration of glutathione in tumor cells is 7–10 times higher than that in normal cells, which in turn, is 100–1000 times higher than that of the extracellular milieu. Hence, glutathione disulfide is considered as an ideal vector‐triggering factor for gene therapy.^[^ ^113^ ^]^ For redox‐responsive delivery systems, the most important and most widely used redox‐sensitive connector is the disulfide bond. This bond is stable in the blood circulation or extracellular environment with low reduction conditions, but is reduced to sulfhydryl groups under high reduction conditions.^[^ ^114^ ^]^ After DNA is coated by PEI connected to the disulfide bond, the DNA is released as the disulfide bond breaks upon reduction by glutathione within the target cells.^[^ ^115^ ^]^ Although this vector has good extracellular stability, the PEI used for coating is cytotoxic. Modification of PEI with PEG or chitosan not only reduces toxicity but also increases transfection efficacy.^[^ ^116^ ^]^ Apart from PEI, liposomes and cationic polymers have been used experimentally to prepare siRNA‐containing redox‐sensitive complexes.^[^ ^117^ ^]^

A synthetic glutathione nanocarrier containing imidazole residues and a disulfide bond‐containing the cationic block copolymer, poly(aspartic acid‐(2‐aminoethyl disulfide)‐(4‐imidazole carboxyl acid))‐PEG, has been demonstrated in vitro to deliver DNA, mRNA and Cas9/sgRNA ribonucleoprotein. The nanocarrier has relatively high transfection efficacy and low cytotoxicity.^[^ ^61^ ^]^ Another cationic polymer containing disulfide bond and imidazole group in the backbone also showed efficient encapsulation, cellular uptake, and endosomal/lysosomal escape. The polymer may be conjugated with amantadine and *β*‐cyclodextrin to further enhance its stability. Compared with the commercially available lipofectamine 2000, a broad‐spectrum transfection agent, the cationic polymer nanocarrier has better gene transfection and gene‐editing efficiency as well as better biocompatibility.^[^ ^118^ ^]^

Calcium phosphate nanoparticles been extensively investigated as a nonviral, inorganic gene delivery system because of their excellent transfection efficacy and biocompatibility. However, uncontrollable crystal growth and the lack of tissue specificity significantly limit their clinical application.^[^ ^119^ ^]^ This problem may be partially solved by combining calcium phosphate with anionic lipid dioleoylphosphatydic acid.^[^ ^120^ ^]^ A core–shell redox‐responsive delivery system has been synthesized that consisted of a positively charged core of calcium phosphate nanoparticle and stimulus‐responsive siRNA, and an anionic shell that consisted of disulfide crosslinked hyaluronic acid that stabilized the calcium phosphate core. The delivery system was stable both in the storage medium and in systemic circulation. The core–shell delivery system successfully released siRNA into the cytoplasm in vitro and in vivo through glutathione‐triggered disassembly and endosomal escape. The hybrid delivery system achieved 80% in vitro gene silencing efficacy. Silencing of the B‐cell lymphoma 2 gene (*Bcl2*) resulted in apoptosis of B16F10 cells. Equipped with the tumor‐targeting hyaluronic acid component, the nanoparticles significantly suppressed the growth of B16F10 xenograft‐induced melanoma tumor in mice. The anionic nanoparticles demonstrated no apparent toxicity in vitro or in vivo, thereby overcoming the inadequacies associated calcium phosphate‐based delivery.^[^ ^121^ ^]^ Other inorganic nanoparticles such as mesoporous silica nanoparticles may also be used as a redox‐responsive vector for gene therapy by conjugating them to poly‐amino esters via disulfide bonds.^[^ ^122^ ^]^

A Fe^2+^‐coordinated supramolecular vesicle which has a reversible fusion–fission function triggered by redox response has been synthesized. When oxidized, the vesicles fuse to form large vesicles. Conversely, when the vesicle is reduced, it splits into smaller vesicles. When loaded experimentally with siRNA, these redox‐responsive vesicles may be uptaken by HeLa cells via endocytosis; the siRNA is subsequently released into the cytoplasm of the HeLa cells.^[^ ^123^ ^]^

Redox‐responsive vectors also promote intracellular delivery of drugs and genes into cancer cells to enhance their antitumor effect. For example, a nanocarrier containing a redox‐sensitive prodrug, 10‐hydroxycamptothecin, and amphiphilic lipids were coated with *Bcl‐2* siRNA and delivered to liver cancer cells. Upon exposure to high concentrations of intracellular glutathione, the released drug and gene inhibited tumor growth by inducing tumor cell apoptosis through the antitumor activity of 10‐hydroxycamptothecin and silencing of the *Bcl‐2* proto‐oncogene.^[^ ^124^ ^]^ Gene therapy delivered by redox‐responsive nanocarriers is often used for treating other diseases. A glutathione‐cleavable nanocomplex was produced by conjugation of DNA‐doped gold nanoparticles with SOX9 siRNA via disulfide bonds. The nanocomplexes were cleaved by glutathione to release SOX9 siRNA to guide neural stem cell differentiation.^[^ ^125^ ^]^ A redox‐sensitive nanoparticle consisting of heparin sodium‐bridged disulfide bond can deliver miRNA and pDNA successfully. Such a system is potentially useful in the rehabilitation of myocardial infarction.^[^ ^126^ ^]^

**Figure 7:**
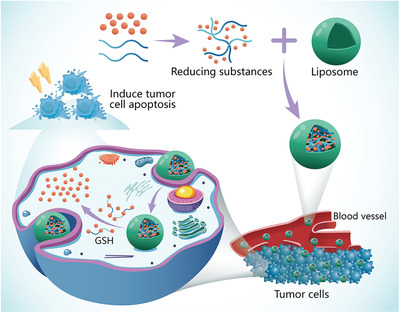
Redox‐responsive nanocarriers for gene therapy. A nanocarrier containing a redox‐sensitive prodrug and amphiphilic lipids were coated with siRNA and delivered to cancer cells. Upon exposure to high concentrations of intracellular glutathione, the released drug and gene inhibit tumor growth by inducing tumor cell apoptosis through the antitumor activity of 10‐hydroxycamptothecin and silencing of the proto‐oncogene.

The use of redox‐responsive vectors requires a high concentration of reducing substances, which may not be present in some diseased tissues (**Figure** **7**). Reduction of disulfide bonds is slower in the mild acidic conditions under which endocytosis occurs. The following issues also deserve attention: 1) most studies are still conducted experimentally at the cellular level; special attention should be paid to testing the in vivo stability of disulfide bonds; 2) because of the complexity of the bond environment, the most favorable conditions that trigger disulfide bond degradation in tumor cells are still not clear; 3) the reductive property of disulfide bond may be adversely affected by steric hindrance of a nanocarrier.^[^ ^12^ ^]^

#### 4.1.3 Enzyme‐Responsive Nanocarriers

Enzymes are an important category of biological stimuli as well as crucial components of metabolic processes. They are highly selective and specific, acting only on selected substrates and active under very mild conditions.^[^ ^127^ ^]^ The concentrations of many extracellular enzymes such as hyaluronidase, matrix metalloproteinases (MMPs), and proteolytic enzymes are elevated in tumorous tissues. High levels of these enzymes can improve uptake by tumor cells. This spurs the development of nanocarrier delivery systems that respond to differences in enzyme concentration.^[^ ^128^ ^]^ However, development of enzyme‐responsive polymer components, nanoparticles, and hydrogels is still in its infancy, compared with other more established stimulus‐responsive gene delivery systems.

The cargo release mechanisms of enzyme‐responsive nanocarriers may be classified as physical or chemical. In systems that function via physical mechanisms, therapeutic agents are released based on enzyme‐catalyzed changes occurring on the surface of nanoparticles. During this process, the enzymatic reaction does not degrade the actual structure of the nanoparticles, but changes the function of the surface, thereby releasing the cargo. In systems that function via chemical mechanisms, the nanocarriers are synthesized in such a way that their structures are sensitive to specific enzymes that degrade the nanocarriers.^[^ ^129^ ^]^

**Figure 8:**
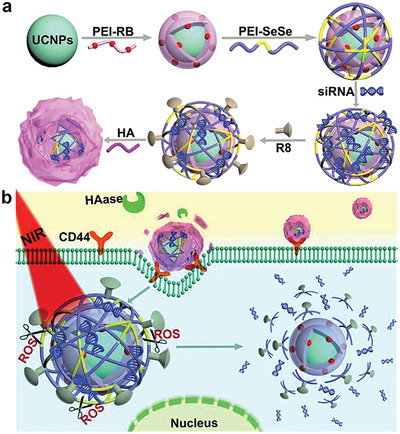
A multilayer nanoparticle siRNA delivery system that utilized hyaluronic acid to disintegrate the outer layer of the hyaluronic acid‐coated nanoparticle to enable other stimuli (near‐infrared irradiation and reactive oxygen species) to continue degrade the nanoparticle for siRNA release. a) The multilayer nanoparticles were composed of cores of upconversion nanoparticles (UCNPs). Each core was functionalized with an inner coating layer of rose bengal (RB) photosensitizer conjugated to polyethylenimine (PRI), a middle coating layer of singlet oxygen (^1^O2)‐sensitive diselenide‐linked PEI (PEI–SeSe) with therapeutic siRNA loading and cell‐penetrating peptide R8 modification, and an outer coating layer of negatively charged hyaluronic acid (HA). b) HA prevented siRNA leakage during delivery process and specifically targeted tumor cells with overexpressed CD44 membrane receptors. The HA outermost coating was first digested extracellular hyaluronidase (HAase) secreted by the tumor cells. Upon the subsequent near‐infrared light (NIR) irradiation at 808 nm, the UCNP core generates emissions at around 540 nm, which activated RB to generate reactive oxygen species (ROS). The latter triggered degradation of the middle PEI–SeSe coating. The NIR light triggered complete decomposition of the UCNO cores, which induced fast and efficient siRNA release for gene silencing and tumor growth suppression. Reproduced with permission.^[^ ^137^ ^]^ Copyright 2019, Elsevier.

Hyaluronic acid is a hydrophilic natural anionic glycosaminoglycans. It is profusely distributed in connective, epithelial, and neural tissues and plays an important role in a variety of pathologies including chronic inflammation, diabetes, and cancer.^[^ ^130^ ^]^ Hyaluronidase is a family of enzymes that degrade hyaluronic acid. Hyaluronidase is involved in the progression of diseases such as cancer, rheumatoid arthritis and traumatic brain injury.^[^ ^131^ ^]^ Pretreatment with hyaluronidase improves the transfection efficiency of pDNA in rat skeletal muscle.^[^ ^132^ ^]^ The most common hyaluronic acid receptor, CD44, is copiously concentrated on the membranes of inflammatory cells and cancer cells, thereby contributing to active delivery.^[^ ^133^, ^134^ ^]^ Hyaluronic acid is a commonly adopted for the construction of hyaluronidase‐responsive gene carriers. Hyaluronic acid prevents leakage of siRNA^[^ ^135^ ^]^ or pDNA^[^ ^136^ ^]^ during delivery and specifically targets tumor cells with overexpressed CD44 membrane receptors. For example, upconversion nanoparticles with multiple layers (nano‐onions) were coated with hyaluronic acid as the outermost layer. Within the tumor microenvironment, the outer layer of hyaluronic acid was enzymatically degraded by the excessive hyaluronidase secreted by the tumor cells. Once the hyaluronic acid‐denuded nanoparticles were attached to the CD44 cell receptors and uptaken into the intracellular milieu, other mechanisms of the multiple stimuli‐responsive system (see Section 4.3) triggered complete disintegration of the nanoparticle that ultimately resulted in the release of the gene cargo (**Figure** **8**).^[^ ^137^ ^]^ By binding to cells that overexpress specific cell surface CD44 receptors, nanomaterials based on hyaluronic acid can actively target diseased tissues that contain these receptors. These nanomaterials are subsequently degraded by hyaluronidase.^[^ ^138^ ^]^

In another example, nanoparticles assembled from hyaluronic acid and calcium phosphate effectively delivered siRNA that targeted epidermal growth factor receptors on the surface of A549 adenocarcinomic human alveolar basal epithelial cells. The hyaluronic acid‐based coating was similarly digested by extracellular hyaluronidase. After cell entry via CD44‐mediated endocytosis, the internalized nanoparticles were responsive to the low pH‐environment of the acidic lysosomes and released siRNA that significantly downregulated epidermal growth factor receptor expression by the A549 cells.^[^ ^139^ ^]^

Tumors are surrounded by an extracellular matrix that contains higher concentrations of crosslinked hyaluronic acid than healthy cells. This is one of the microanatomical barriers that impede effective antitumor drug and gene delivery.^[^ ^140^ ^]^ A hyaluronidase‐based nanoparticle drug/gene release system has been synthesized by modification of hyaluronidase with dextran. The hyaluronidase‐dextran nanoparticles demonstrated enzyme stability, reduced immunogenicity, and prolonged blood half‐life after intravenous injection. The nanoparticles were pH‐responsive and dissociated within the tumor acidic environment to release hyaluronidase. The latter catalyzed the breakdown of hyaluronic acid to loosen the extracellular matrix. This, in turn, enabled oxygen penetration and release of the therapeutic cargo. The relieved tumor hypoxic condition also improved the efficacy of subsequent photodynamic therapy (PDT) and augmented the effect of antitumor immunotherapy.^[^ ^141^ ^]^

Matrix metalloproteinases (MMPs) are associated with cancer invasion, progression, and metastasis. A synthetic octapeptide (GPLGIAGQ) was used as a stimulus‐sensitive connector in micellar nanocarriers for MMP‐2‐triggered targeted antitumor therapy. Compared with nonresponsive complexes, MMP‐2 sensitive micelles may be used as gene delivery systems to implement strategies that enhance drug permeability and improve treatment efficacy.^[^ ^142^ ^]^

#### 4.1.4 Other Biologically Responsive Nanocarrier Schemes

Apart from the aforementioned stimulus‐responsive delivery systems, other biologically responsive systems include reactive oxygen species (ROS), adenosine triphosphate (ATP), as well as hypoxia‐ and inflammation‐responsive nanocarriers.

Reactive oxygen species refer to highly active molecules or free radicals present in cells. They include hydrogen peroxide (H_2_O_2_), singlet oxygen (^1^O_2_), superoxide (O^−^_2_), and hydroxyl radical (^•^OH), which are important molecules in signal transduction and metabolism.^[^ ^143^ ^]^ A plethora of pathological diseases, including cancer, inflammation and cardiovascular diseases, are associated with high intracellular ROS levels. Thus, ROS may be used as a stimulus for gene delivery.^[^ ^137^, ^144^ ^]^ Reactive oxygen boron carriers in lipid envelope exhibit potent sensitivity to intracellular H_2_O_2_ and may be used to deliver siRNA for combating cancer.^[^ ^145^ ^]^ Unlike traditional polymers, supramolecular polymers combine the advantages of supramolecular chemistry and polymers. Supramolecular polymers possess specific properties such as degradability and susceptibility to stimulation, based on dynamic reversible noncovalent interactions.^[^ ^146^ ^]^ A range of materials containing boric acid ester^[^ ^147^ ^]^ or peroxalate ester^[^ ^148^ ^]^ can respond to reactive oxygen microenvironments. Oxidative‐responsive supramolecular polycation assemblies (CPAs) deliver nucleic acids via polycations based on *β*‐cyclodextrin. Reactive oxygen species accelerate the decomposition of CPA/pDNA complexes and promote the release of pDNA from those complexes to facilitate transfection.^[^ ^149^ ^]^ Poly[(2‐acryloyl)ethyl(*p*‐benzyl borate)diethyl ammonium bromide] undergoes positive–negative charge conversion under high intracellular ROS concentration. When the H_2_O_2_ level in tumor cells increases, the boric acid group is oxidized by the ROS. After cationic lipid modification, the modified nanocarrier has improved serum stability, more effective cell uptake and lysosomal escape, and is more effective for gene therapy.^[^ ^150^ ^]^

Adenosine triphosphate is one of the most important biomolecules for cellular energy metabolism. Its intracellular concentration is much higher than that present in the extracellular milieu. This is the rationale behind the design of ATP‐responsive gene delivery vectors.^[^ ^151^ ^]^ In one example, PEG was used to block cationic polyplex micelles derived from 4‐carboxyl‐3‐fluorophenylboronic acid. d‐gluconamide was used to facilitate de‐condensation of pDNA, which was triggered by increased intracellular ATP concentration and enhanced gene transfection.^[^ ^152^ ^]^ Triton X‐100 is a nonionic detergent. Because its high transmembrane capacity, the uptake of siRNA by cells and the escape of siRNA from endosomes/lysosomes are greatly improved in its presence. Triton X‐100 modified ATP‐responsive transfection reagent effectively transferred siRNA into cancer cells, and demonstrated significant tumor growth inhibition in murine tumor models.^[^ ^153^ ^]^ Compared with the traditional scheme of controlling nucleic acid release in the cytoplasm via trigger‐induced polycation degradation, the combination of charge transformation and degradation represents a new strategy in gene delivery. Polycations prepared by crosslinking of phenylboronic acid‐grafted PEI with alginate are capable of disassembly and charge reversal when triggered by intracellular ATP. This resulted in efficient and rapid release of siRNA by electrostatic repulsion.^[^ ^154^ ^]^ ATP‐responsive zeolite imidazole framework‐90 serves as a delivery platform for cytosolic CRISPR/Cas9 genome editing, regardless of the size or molecular weight of the protein.^[^ ^155^ ^]^ ATP‐responsive vectors may also be used for the coordinated delivery of chemotherapeutic drugs and pDNA^[^ ^156^ ^]^ or miRNA.^[^ ^157^ ^]^

Because tumor lesions often have anoxic regions, it is possible to use hypoxia‐responsive nanomaterials to construct nanoplatforms for targeted tumor therapy.^[^ ^158^ ^]^ Commonly used hypoxia‐sensitive materials include 2‐nitroimidazole^[^ ^159^ ^]^ and azobenzene complexes.^[^ ^160^ ^]^ Amphiphilic polycations formed by coupling of hydrophobic 2‐nitroimidazole with alkylated PEI are converted to hydrophilic 2‐aminoimidazole under hypoxic conditions through biological transformation. This not only helps to improve the stability of the polymer complex and increase cell uptake, but also promotes the dissociation of siRNA in the cytoplasm and ultimately improves gene silencing efficacy.^[^ ^161^ ^]^ Copper‐based metal–organic nanoparticles have good stability under normal oxygen pressure. Upon exposure to a hypoxic tumor microenvironment, the nanoparticles are rapidly degraded to release Cu^2+^ and chlorin e6. This significantly enhances the penetration depth of the nanoparticles within the tumor and offers a novel approach for the design and development of hypoxia‐responsive nanoplatforms for diagnosis and treatment.^[^ ^162^ ^]^

Another strategy available is the inflammatory response‐mediated system. Inflammatory responses are mediated by immune cells such as T lymphocytes and B lymphocytes. Using modified calcium chloride‐mediated transformation, siRNA and miRNA mimics or inhibitors incorporated in serum‐derived exosome carriers have been successfully delivered to lung macrophages via intratracheal instillation.^[^ ^163^ ^]^

### 4.2 External Environmentally Responsive Nanomaterials

External environmentally responsive vectors control the release of nucleic acids spatially and temporally. For example, a light‐responsive gene delivery system controls gene release through photoperiod splitters as long as the light is illuminated at a specific wavelength.^[^ ^164^ ^]^ In the subsections that follow, more examples of exogenous triggers for gene delivery will be reviewed.

#### 4.2.1 Temperature‐Responsive Nanocarriers

**Figure 9:**
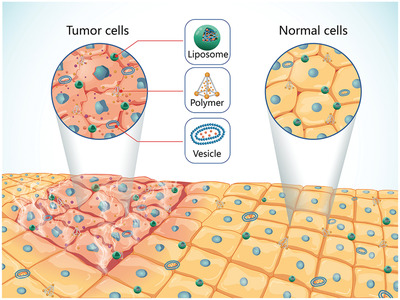
Temperature‐responsive nanocarriers for gene therapy. Tumor tissue is slightly warmer than normal tissue. When the temperature increases, vectors including liposomes, polymeric micelles, and vesicles become soluble and release the co‐delivered anticancer drugs.

Temperature may be used as an external stimulus when heat is applied externally, or as an internal stimulus when the temperature of a pathological region rises naturally. Tumor tissue is slightly warmer than normal tissue. Heating the tumor site increases the local blood supply and permeability of the tumorous tissues. Temperature‐responsive vectors including liposomes, polymeric micelles and vesicles are used extensively in combination with other stimuli (**Figure** **9**).^[^ ^165^ ^]^ Among these vectors, polymer hydrogels are the most commonly used temperature‐responsive nanomaterials. The properties of the polymers enable them to have a critical solution temperature range, which the polymer becoming insoluble when the temperature increases beyond the lower critical solution temperature.^[^ ^13^ ^]^

Poly(*N*‐isopropyl acrylamide) (PNIPAAm) is the most popular thermosensitive polymer used for gene delivery.^[^ ^166^ ^]^ Liposomes modified with the thermo‐responsive polymer P(NIPAAm‐*co*‐*N*,*N*′‐dimethylaminopropyl acrylamide) have a lower critical solution temperature comparable with that of pristine liposomes, with excellent intracellular gene silencing effects.^[^ ^167^ ^]^

Another common type of temperature‐responsive carrier is thermosensitive liposomes. The transition temperature (Tm) of phospholipids from the gel phase to fluid phase is the strategic parameter of thermosensitive liposomes. When the temperature is higher than Tm, the fluidity and permeability of the thermosensitive liposome increase, and the cargo is released quickly and effectively. For liposomes to be effective for drug or gene delivery, the Tm of liposomes should be slightly higher than 37 °C.^[^ ^168^ ^]^ Thermosensitive cationic liposomes that co‐deliver anticancer drugs (doxorubicin or quercetin) and CDC20 siRNA have notable inhibitory effect on gastric cancer cells.^[^ ^169^ ^]^

Temperature‐responsive vectors are often used in conjunction with other stimulus, which are described in subsequent sections on multiple stimuli‐responsive nanocarriers. Thermosensitive materials do have limitations. For example, thermal triggering of some nanocarriers cannot be controlled satisfactorily. The biodegradability of pristine PNIPAAm is not satisfactory, which limits its applications in clinical medicine; poly(*ε*‐caprolactone) and/or PEG have to be incorporated into PNIPPAAm thermoresponsive hydrogels to enhance their biodegradability.^[^ ^170^ ^]^ Improving the biodegradability of thermal‐sensitive materials will be the future development direction.

#### 4.2.2 Light‐Responsive Nanocarriers

Light is frequently used, in various wavelengths, as triggers for the release of genetic materials from nanocarriers. Light may be precisely controlled spatially and temporally for the release of drugs or nucleic acids. Common nanocarriers such as polymers, cationic liposomes or gold nanoparticles may be used for the delivery of light‐sensitive nucleic acids. Several strategies have been used to produce light‐activated nanoparticle delivery systems. The most common strategy is to incorporate light‐dissociable molecules such as *o*‐nitrobenzyl or azobenzene into the polymer backbone.^[^ ^171^ ^]^ The structure of light‐sensitive nanocarriers can be changed directly or indirectly to release cargo under light activation. There are three mechanisms of cargo release: 1) morphological transformation of nanocarrier induced by photoisomerization; 2) degradation of nanocarrier caused by light reaction; 3) photothermally induced nanocarrier rupture.^[^ ^172^ ^]^

Ultraviolet light (UV; 10–400 nm), visible light (400–750 nm), or near‐infrared light (NIR; 750–900 nm) may be used as the stimulus.^[^ ^173^ ^]^ Although UV light has a higher energy and produces more proficient photochemical reactions, prolonged exposure to UV light may cause damage to the body because it is easily absorbed by hemoglobin, lipids and water and does not penetrate deep into body tissue.^[^ ^174^ ^]^ Conversely, NIR light is less energetic but penetrates deeper into body tissue and is more compatible with cells.^[^ ^175^ ^]^ By using upconversion nanoparticles as an optical converter, the NIR light that passes through body tissue may be converted to visible light. The NIR light outside body tissue is not easily scattered, whereas the visible light inside the tissue can effectively control the switch of ion channel. This strategy has potential to stimulate deep brain neurons for the treatment of Parkinson's disease.^[^ ^176^ ^]^ A recent study reported that siRNA release from silica core/gold shell core–shell nanostructures under pulsed laser or continuous wave NIR irradiation is a temperature‐independent process. The efficacy of siRNA release under pulsed irradiation is much higher than under continuous wave irradiation.^[^ ^177^ ^]^ The problem that the NIR photon energy is too low to undergo photochemical reactions may be solved by the mechanism of two‐photon absorption or upconversion. Visible light is mainly used for superficial application on skin and mucous membranes.^[^ ^178^ ^]^ Ultraviolet light, visible light, or high‐power density NIR light may cause undesirable thermal damage. Far‐red light also mediates the delivery of antitumor genes.^[^ ^179^ ^]^ A co‐delivery system consisting of ROS degradable polycations and photosensitizers enables the delivery of programmable genes using far‐red light at low optical power density.^[^ ^180^ ^]^

**Figure 10:**
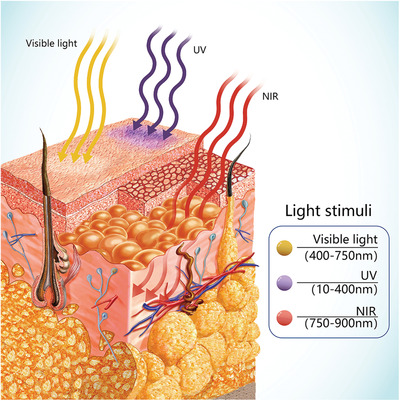
Light‐responsive nanocarriers for gene therapy. Light stimuli include ultraviolet light (UV; 10–400 nm), visible light (400–750 nm), and near‐infrared light (NIR; 750–900 nm). Ultraviolet light has a higher energy and produces more proficient photochemical reactions. Near‐infrared light is less energetic but penetrates deeper into body tissue and is more compatible with cells.

Light sources may also be used for other nanomedicine therapies. Photochemical reactions of photosensitizers under specific wavelength produce oxygen free radicals or heat energy to damage target cells, thereby selectively killing the diseased cells (**Figure** **10**). Such treatments are known as PDT and photothermal therapy (PTT).^[^ ^181^ ^]^ Photochemical internalization is an evolutionary technique for PDT that offers a strategy for triggering endosomal/lysosomal escape. Photochemical internalization has been employed to deliver a variety of drugs and biological molecules. However, the complicated synthesis involved in self‐assembly of photosensitizers, cargos and polymers, as well as the restrictive cargo loading requirements limit its application in gene delivery.^[^ ^182^ ^]^ A highly fluorescent graphitic hollow carbon nitride nanosphere (GHCNS) was used as a nanocarrier and photosensitizer by having its surface modified with hyaluronic acid. Upon visible light irradiation, the GHCNS produces ROS, effectively induces lipid peroxidation, causes endosomal/lysosomal membrane rupture and expedites the release of the loaded cargo into the cytoplasm of target cells.^[^ ^183^ ^]^

#### 4.2.3 Other Environmentally Responsive Nanocarrier Schemes

Other environmentally responsive stimuli for gene therapy include ultrasonic energy^[^ ^184^ ^]^ application of a magnetic field^[^ ^185^ ^]^ or application of an electrical field.^[^ ^186^ ^]^

Ultrasonic signals may be reflected, deviated or absorbed depending on the environment. Their advantages include high efficiency, safety and low invasiveness. Ultrasound‐responsive gene delivery systems have been extensively studied for treatment of cancer and neurodegenerative diseases.^[^ ^187^ ^]^ The combination of ultrasonic stimulation and microvesicles has demonstrated great potential for intracellular gene delivery.^[^ ^188^ ^]^ Sound penetration by microvesicles is thought to contribute to increased gene uptake via two potential mechanisms: first, cell membrane deflection induces endocytosis, and second, microvesicle ejection induces the formation of pores in the cell membrane, which enhances localized gene delivery.^[^ ^189^ ^]^

Ultrasound‐mediated gene transfer usually needs to be completed under a high peak negative pressure (PNP) of more than 2 MPa. However, more recent studies showed that such a high pressure may not be necessary to induce cavitation (implosion of microbubbles).^[^ ^190^ ^]^ Microbubbles generated by ultrasound have microscale and polydisperse particle size distribution. It is difficult for the microbubbles generated by ultrasonic cavitation to come in contact with tumor cells and deliver therapeutic genes. Recently, a novel cationic biosynthetic nanobubble was extracted from *Halobacterium* NRC‐1, which has a protein shell of about 200 nm. This nanobubble is hollow and allows dissolved gas to transfer freely. When coated with PEI, the nanobubble demonstrated improve DNA loading capacity. Upon ultrasonic activation, gene transfection efficacy into tumor cells was significantly improved.^[^ ^191^ ^]^ Compared with plasmids, viruses and other delivery systems, direct delivery of Cas9/sgRNA protein has the advantages of low nontargeted effect and nonintegration, but still has limitations due to low transfer efficacy. Clustered, regularly interspaced, short palindromic repeats (CRISPR)‐associated protein 9 (Cas9) genome editing is being developed in combination with nanocarrier technology to improve Cas9 transportation efficacy and biocompatibility. A microbubble‐conjugated nanoliposome delivery system has been used as carrier for the Cas9/sgRNA complex. When subjected to ultrasonic activation, the complex was successfully delivered to dermal papilla cells of hair follicles of animals with androgenetic alopecia (male‐pattern baldness) through acoustic perforation of particles by microbubble cavitation. The targeted 3‐oxo‐5‐alpha‐steroid 4‐dehydrogenase 2 (*SRD5A20*) gene, which is responsible for the pathogenesis of male‐pattern baldness, was specifically recognized and efficiently edited in vivo, thereby inhibiting the production of the SRD5A2 protein.^[^ ^192^ ^]^

The major challenge of effective gene therapy for central nervous system diseases is the presence of the blood–brain barrier (BBB). Conventional intracranial injection produces poor gene expression and may cause nerve damage. In addition, the ability of virus vectors to cross the BBB is poor. Ultrasound‐mediated gene therapy can solve this problem.^[^ ^193^ ^]^ Focused ultrasound may be used for local BBB opening and gene delivery.^[^ ^194^, ^195^ ^]^ For example, ultrasound‐mediated *Bone Morphogenetic Protein‐6* gene transfer to endogenous mesenchymal progenitor cells expedited healing of functional fractures and ligaments.^[^ ^196^ ^]^

Although ultrasound‐triggered gene transfection systems have many advantages, development of ultrasound‐responsive nanoparticles for gene delivery is hampered by the absence of a clear understanding of the mechanism of acoustic cavitation and perforation. Nevertheless, ultrasound has great development potential as a future tool to improve gene transfection efficacy.

Because a magnetic field has almost no physical interaction with the body, it is one of the best choices for external physical stimulation. Nanoparticles such as iron oxide need to contain the magnetic metal elements in order to be manipulated by a magnetic field. However, a single superparamagnetic nanoparticle cannot carry nucleic acid. Surface modification with polymer, liposome or inorganic metal provides opportunities for magnetic nanoparticle‐guided gene transfection.^[^ ^197^ ^]^ By coating magnetic silica nanoparticles with tannic acid, functional siRNAs were successfully introduced into the cytoplasm of human osteosarcoma cells in vitro.^[^ ^198^ ^]^ Shielding of surface charges by PEGylated magnetic nanoparticles improved the transfection efficacy of targeted hippocampal neurons and reduced nanoparticle neurotoxicity.^[^ ^199^ ^]^ A folate receptor‐targeted magnetic mesoporous silica nanoparticle has been developed for joint delivery of vascular endothelial growth factor‐short hairpin RNA (shRNA) and doxorubicin for anticancer treatment.^[^ ^200^ ^]^ Although substantial progress has been achieved lately in magnetic field‐triggered gene transfection, the disparity in biocompatibility continues to hinder translation from benchtop research to bedside applications. Modification of superparamagnetic surface material should produce carrier systems with good biocompatibility and efficient genetic load capacity. Bionic materials are expected to overcome the biosafety problems of organic polymers.

Electric fields have the feasibility of precise control or telecontrol. Although electric fields are used extensively for on‐demand drug delivery, they are rarely used for gene transfection.^[^ ^201^ ^]^ Controlled and targeted delivery of genes into cells and living animals may be realized by electrically activated plasmonic Au nanoparticles.^[^ ^202^ ^]^ Nucleic acids, functional proteins and Cas9 unidirectional RNA proteins may be delivered by a nanopore electroporation platform to adherent cells and suspension cells.^[^ ^186^ ^]^ Transdermal gene delivery through a combination of electroporation arrays enables safe and convenient gene expression and siRNA transfection.^[^ ^203^ ^]^ **Table** **3** summarizes the advantages and disadvantages of different types of stimulus‐responsive nanocarriers for gene therapy.

**Table 3:** Advantages and disadvantages of stimuli‐responsive gene delivery system. Abbreviation: PNIPAAM, Poly(*N*‐isopropylacrylamide)

Stimulus‐responsive gene delivery system	Advantages	Disadvantages	Refs.
pH	pH responsive; stability in blood circulation; enhanced cellular uptake; low cytotoxicity	Poor specificity because endosomes and lysosomes of all cells are acidic; Changes in pH between diseased tissues and surrounding normal tissues may not be very significant; Different types of diseased tissues may have slightly different pH levels	^[^ ^89^, ^94^, ^95^, ^96^, ^97^, ^204^ ^]^
Redox	High extracellular stability; responds quickly and releases genes quickly inside the cell	Lack of specificity and precision for differences in intracellular and extracellular concentrations of GSH in both normal and tumor cells; Non‐selective response delivery may lead to unexpected release and related side effects; The reductive property of disulfide bond may be adversely affected by steric hindrance of a nanocarrier	^[^ ^12^, ^119^ ^]^
Enzyme	Enzymatic reaction is rapid and reaction conditions are mild; enzymes are selective to substrates with high selectivity and specificity	Development of enzyme‐responsive polymer components, nanoparticles and hydrogels is still in its infancy; Enzymes at nontarget sites or similar substrates of the same family of enzymes may also trigger cargo release	^[^ ^127^ ^]^
ROS	Responds according to different ROS levels in the tissue	Biocompatibility, accuracy and sensitivity of stimulus response need to be further improved; In vivo research is still in its infancy	^[^ ^142^ ^]^
ATP	ATP exists in both intracellular and extracellular tissues, intracellular concentration is much higher than extracellular concentration particularly in tumor cells	Nonspecific distribution in entire organ/tissues and lack of spatial resolution	^[^ ^205^ ^]^
Hypoxia	High sensitivity, can target the deep hypoxic region of the tumor	Poor stability; Specificity is not high; Biocompatibility is poor; Synthesis is complex	^[^ ^206^ ^]^
Inflammation	Inflammatory reaction is common	Specificity is not strong; Current studies are rare	^[^ ^163^ ^]^
Temperature	Easy to control and convenient operation, effective transfection	Local high temperature is not always significantly different from normal tissue; The commonly used PNIPAAM may be mildly toxic after prolonged exposure	^[^ ^165^, ^166^, ^167^ ^]^
Light	Response is achieved by external light stimulation; Temporally and spatially controlled with high efficacy	Light penetrates tissue at shallow depths and may cause thermal damage	^[^ ^207^ ^]^
Ultrasonic	High tissue penetration; accurate positioning, safety and low invasiveness	Absence of a clear understanding of the mechanism of acoustic cavitation and perforation; When the tumor size is large, the delivery vector may not be effective due to the heterogeneity of tissue and the non‐homogeneity of sound transmission	^[^ ^189^, ^208^ ^]^
Magnetic	Little physical interaction with the body; nanocarriers may be mediated in a directional manner with safety and effectiveness	Biocompatibility; Genetic load capacity and stability are not high; Inconvenient to use	^[^ ^197^ ^]^
Electrical	Flexible equipment that allows precise remote control	Slow response; Requires a controllable voltage source; Not easy to operate in a physiological environment	^[^ ^202^, ^203^ ^]^

### 4.3 Multiple Stimuli‐Responsive Nanocarriers

**Figure 11:**
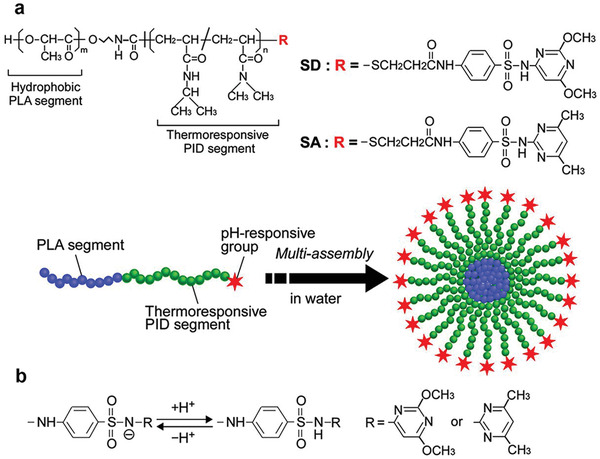
a) Chemical structure of sulfonamide‐terminated block copolymers used to form pH‐ and temperature‐responsive micelles. The micelles were formed by self‐assembly of polymers through aqueous dialysis and possessed a hydrophobic core, temperature‐responsive corona, and a pH‐responsive sulfonamide group. Two types of sulfonamides (SD and SA) possessing different pKa values were tested using mildly acidic conditions. b) pH‐dependent property changes of the sulfonamide derivative. a,b) Reproduced with permission.^[^ ^210^ ^]^ Copyright 2018, Wiley‐VCH.

Many multiple stimuli‐responsive nanocarriers have been developed to deliver genes more effectively and accurately for combating diseases that are not readily receptive to treatment with single stimulus‐responsive gene carriers.^[^ ^209^ ^]^ Multiple stimuli‐responsive systems utilize more than one stimulus responses. For example, pH/temperature‐responsive nanocarriers were prepared from two sulfonamide‐functionalized poly(*N*‐isopropylacrylamide)‐based polymeric micelles (**Figure** **11**).^[^ ^210^ ^]^ When loaded with a proof‐of‐concept antiproliferative drug, both sulfadimethoxine surface‐functionalized and sulfamethazine surface‐functionalized micelles demonstrated enhanced intracellular uptake under mildly acidic conditions (pH 6.8) at temperatures slightly above their lower critical solution temperatures. Both micelle variations possessed the capability to be used as an intracellular pH/temperature responsive drug or gene delivery system.

**Figure 12:**
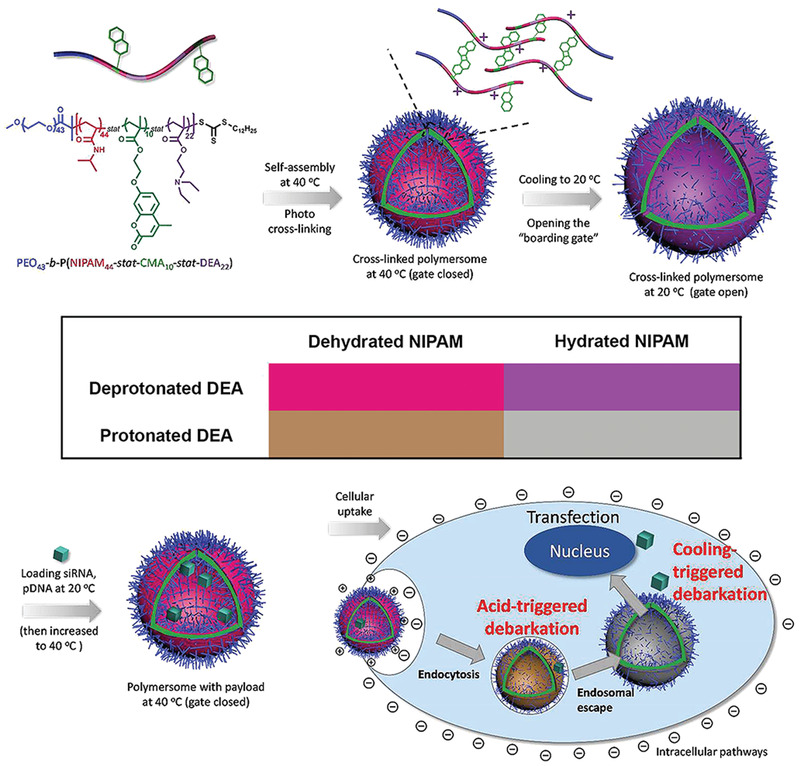
Schematic of a dually gated polymersome as a bio‐macromolecular shuttle for gene delivery. The crosslinked polymersome with a “boarding gate” and a “debarkation gate” can load biomacromolecules by opening the “boarding gate” at room temperature, can lock them at body temperature in pure water, and can release them through the “debarkation gate” at acidic conditions such as in endosomes at physiological temperature. The release of biomacromolecules can be further accelerated by enabling the initial “boarding gate” as a second “debarkation gate” upon slight decreasing in temperature within the target cells. The colors of the bars in the center of the schematic represent different states of the polymersome membrane depending on the temperature and pH. Abbreviations: NIPAM, *N*‐isopropylacrylamide; DEA, 2‐(diethylamino)ethyl methacrylate. Reproduced with permission.^[^ ^211^ ^]^ Copyright 2018, American Chemical Society.

In another example, a pH/temperature‐responsive, dual‐gated polymersome was self‐assembled from a block copolymer, poly(ethylene oxide)‐block‐poly[*N*‐isopropylacrylamide‐stat‐7‐(2‐methacryloyloxyethoxy)‐4‐methylcoumarin‐stat‐2‐(diethylamino)ethyl methacrylate] (PEO‐*b*‐P(NIPAM‐stat‐CMA‐stat‐DEA).^[^ ^211^ ^]^ This polymersome possessed a “boarding gate” and a “debarkation gate” in the membrane to provide a gating system for efficient loading, protection, delivery and release of environmentally sensitive nucleic acids on demand (**Figure** **12**). The hydrophilic PEO chains formed the coronas of the polymersome, whereas the pH‐ and temperature‐sensitive P(NIPAM‐stat‐CMA‐stat‐DEA) block copolymer formed the dual‐gated heterogeneous membrane. The temperature‐controlled “boarding gate” could be opened at ambient temperature for siRNA and pDNA encapsulation. The “debarkation gate” could be triggered by a proton sponge effect (i.e., the presence of a weakly basic molecule may cause an endosome to burst) for intracellular release to enable efficient in vitro and in vivo gene transfection. Together with the previous example, these strategies open new vistas in the design of multiple stimuli‐responsive nanocarriers for gene delivery.

**Figure 13:**
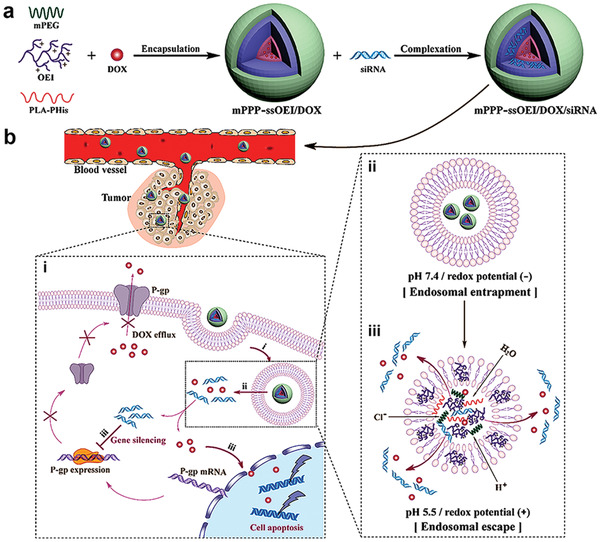
a) Schematic of a pH/redox dual‐responsive mPPP‐*ss*OEI/DOX/siRNA co‐delivery polyplex with effective endosomal–lysosomal escape. b) Schematic of the multidrug‐resistant reversal of the polyplex in vivo. The polyplexes were administered through intravenous injections and accumulated in tumor tissue with an EPR‐mediated targeting effect. After internalization i), the acidic and reduction potential environments of the endosomes‐lysosomes trigger cargo release and subsequent endosomal–lysosomal escape ii). The free siRNA and DOX demonstrated synergy in gene silencing and cell apoptosis to the drug‐resistant tumor cells iii). Abbreviation: DOX, doxorubicin. a,b) Reproduced with permission.^[^ ^214^ ^]^ Copyright 2019, American Chemical Society.

A lot of attention has been devoted by scientists lately to the development of pH and redox dual‐responsive nanocarriers for gene delivery applications. These nanocarriers not only enhance the escape capability of therapeutic nucleic acid from endosomes by using their acid‐responsiveness, but also improve the release efficacy of therapeutic nucleic acid to the cytoplasm by virtue of their reducibility.^[^ ^212^, ^213^ ^]^ A pH‐ and redox‐responsive polyplex with highly effective endosomal/lysosomal escape has been developed for co‐delivery of multi‐drug resistance‐1 (MDR‐1) siRNA and the anticancer drug doxorubicin against drug‐resistant tumor cells.^[^ ^214^ ^]^ This polyplex was synthesized from methoxy‐PEG‐polylactide‐polyhistidine‐*ss*‐oligoethylenimine (mPEG‐*b*‐PLA‐PHis‐*ss*OEI). The siRNA and drug‐encapsulated polyplex triggered cargo release in response to pH and redox stimuli because of phosphohistidine protonation and disulfide bond cleavage (**Figure** **13**).

**Figure 14:**
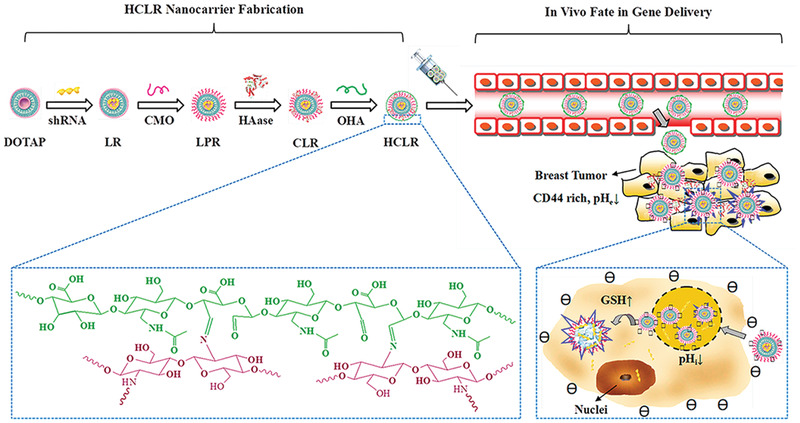
Schematic of the synthesis of a pH‐ and redox‐responsive nanocarrier for delivery of surviving shRNA for silencing the inhibitor of apoptosis by RNA interference technology for in vivo breast tumor targeting gene delivery. Single abbreviations: CS, chitosan; CMO, chitosan with a hydrophobic oleic acid tail; DOTAP, 1,2‐dioleoyl‐3‐trimethylammonium propane; HA, hyaluronic acid; HAase, hyaluronidase; OHA, oxidized hyaluronic acid; pH_e_, pH in the extracellular environment; pH_i_, pH in the intracellular environment. shRNA, short hairpin RNA. Compound abbreviations: LR, DOTAP/survivin‐shRNA; LPR, CS/DOTAP/survivin‐shRNA lipopolyplex; CLR, HAase/CS/DOTAP/survivin‐shRNA; HCLR, HA/HAase/CS/Liposome/survivin‐shRNA. Reproduced with permission.^[^ ^215^ ^]^ Copyright 2020, American Chemical Society.

In another example of pH‐ and redox‐responsive gene delivery, a polysaccharide‐enveloped liposome nanocarrier has been synthesized for RNAi via survivin gene silencing.^[^ ^215^ ^]^ Survivin, an inhibitor of cell apoptosis, is strongly expressed in malignant tumors such as breast cancer and increases of risk of cancer cell proliferation and metastasis. The polysaccharide‐enveloped liposome carrier was constructed by layer‐and‐layer deposition of redox‐sensitive chitosan with a hydrophobic oleic acid tail, and hyaluronic acid onto the liposome. This was followed by sequential loading of survivin‐shRNA and the permeation promoter hyaluronidase. The hyaluronic acid/hyaluronidase/chitosan/liposome/shRNA (HCLR) nanocarrier was stable in the blood circulation because of the negatively charged hyaluronic acid shield. Deshielding of hyaluroniase and chitosan occurred in the slightly acidic extracellular tumor environment, which increased the uptake of the survivin‐shRNA loaded, deshielded liposomes into breast cancer cells. Liposome escape occurred upon reduction in intracellular pH within the endosomes. Release of the survivin‐shRNA was expedited by the high glutathione concentration within the cytoplasm of the tumorous cells. The HCLR system was relative nontoxic to normal murine cells and significantly reduced tumor volume in mice through survivin silencing (**Figure** **14**).

**Figure 15:**
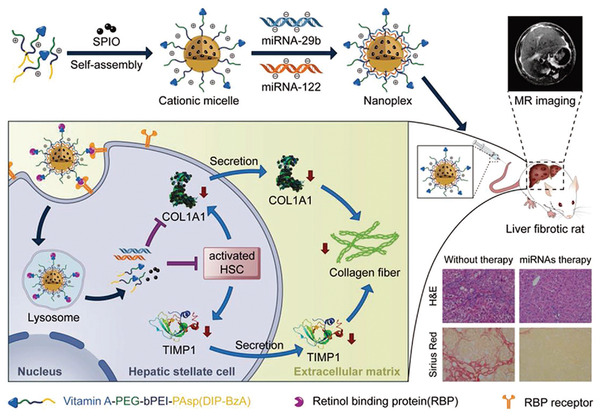
Schematic of the preparation of vitamin A‐decorated pH‐sensitive and SPIO‐loaded nanoplex T‐PBP@miRNA/SPIO (T‐miRNA/S) for targeting delivery of two miRNAs, miRNA 29b and MiRNA‐122. The assembly synergistically downregulated the expression of liver fibrosis‐related genes for alleviating liver fibrosis. Red arrows indicate reduction of COL1A1, TIMP1 and collagen fibrils. Abbreviations: COL1A1, collagen type I alpha 1 protein; TIMP1, tissue inhibitor of metalloproteinase‐1; SPIO, superparamagnetic iron oxide. Reproduced under the terms of the Creative Commons CC‐BY license.^[^ ^216^ ^]^ Copyright 2019, TheAuthors, published by Wiley‐VCH.

A pH‐ and magnetic field‐responsive nanocarrier has been developed for experimental use by synthesizing a pH‐sensitive and vitamin A (VA)‐conjugated copolymer, VA–polyethylene glycol–polyethyleneimine–poly(*N*‐(*N′,N′*‐diisopropylaminoethyl)‐*co*‐benzylamino) aspartamide (T‐PBP). This copolymer was assembled into superparamagnetic iron oxide‐decorated cationic micelles for miRNA delivery.^[^ ^216^ ^]^ The T‐PBP micelles efficiently transported miRNA‐29b and miRNA‐122 to hepatic stellate cells in liver fibrotic rats that were visible using magnetic resonance imaging. The assembly created a synergistic antifibrosis effect via downregulation of fibrosis‐related genes expression (**Figure** **15**). Those downregulated genes included collagen type I alpha 1, *α*‐smooth muscle actin, and tissue inhibitor of metalloproteinase‐1. The dual‐responsive nanocarrier‐miRNA antifibrotic assembly has potential to improve liver function.

**Figure 16:**
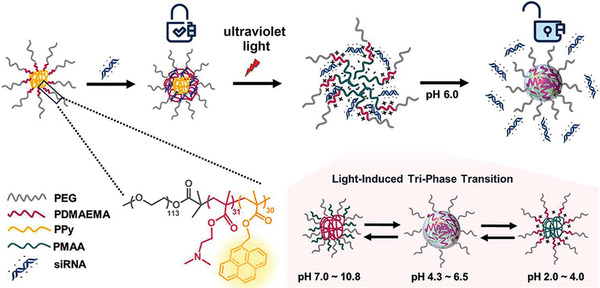
Schematic of binding and release of siRNA from self‐assembled micelleplexes that undergo structural transitions in response to pH and ultraviolet light stimuli. Abbreviations: PEG, polyethylene glycol; PDMAEMA, poly(2‐dimethylaminoethyl methacrylate); PPy, poly(pyrenylmethyl methacrylate); PMAA, poly(methacrylic) acid. Reproduced with permission.^[^ ^217^ ^]^ Copyright 2019, The Royal Society of Chemistry.

pH‐ and light‐responsive micelleplexes have been developed via the synthesis of the triblock copolymer PEG‐*b*‐poly(2‐dimethylaminoethyl methacrylate)‐*b*‐poly(pyrenylmethyl methacrylate). The PEG‐*b*‐PDMAEMA‐*b*‐PPy self‐assembled micelles were used for delivery of miRNA.^[^ ^217^ ^]^ The troblock copolymer was responsive to pH changes because the PDMAEMA segment is a weak electrolyte with pH responsiveness. The micelles expanded upon decrease in pH from physiologic pH to pH 6. The PEG‐*b*‐PDMAEMA‐*b*‐PPy copolymer was also photoresponsive to UV light at 365 nm, which cleaved the ester bond on the PPy block, converting the PDMAEMA to poly(methacrylic acid) (**Figure** **16**). The combination of UV light irradiation and pH change resulted in a burst release of the loaded SiRNA from the micelleplexes upon their uptake by MDA‐MB‐231 human epithelial breast cancer cells.

**Figure 17:**
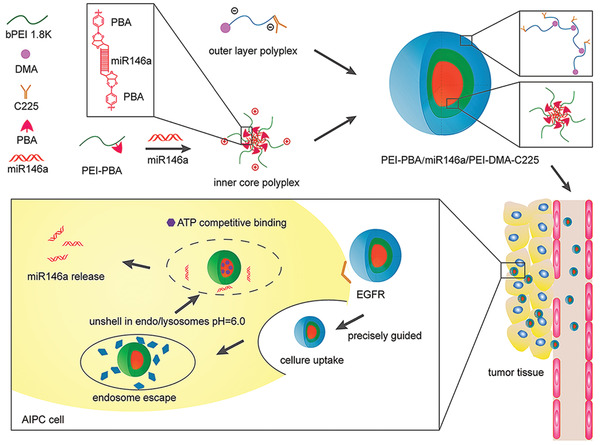
Schematic of the synthesis and micro‐RNA release from pH‐ and ATP‐responsive biolayered polymeric nanocomplex (polyplex). Abbreviations: AIPC, androgen‐independent prostate cancer; ATP, adenosine triphosphate; DMA, dimethylmaleic anhydride; miRNA, micro‐RNA; PBA, 4‐(bromomethyl) phenylboronic acid; PEI, polyethylenimine. Reproduced with permission.^[^ ^218^ ^]^ Copyright 2020, American Chemical Society.

A layered pH‐ and ATP‐responsive nanocomplex has been designed to deliver miRNA (miR146a), to inhibit the expression of epidermal growth factor receptor for the treatment of androgen‐independent prostate cancer (AIPC).^[^ ^218^ ^]^ The complex consisted of an inner core polyplex prepared from poltethylenimine‐4‐(bromomethyl) phenylboronic acid‐miR146a (PEI‐PBA‐MiR146a). The outer layer polyplex, consisting of PEI‐demethylmaleic anhydride‐cetuximab (PEI‐DMA‐C225), was assembled over the inner core polyplex by electrostatic interaction. When uptaken by AICP cells, the outer layer was unshelled in response to the low pH environment (pH 6.0), enabling the inner core to escape into the cytoplasm of the tumorous cells. The high ATP concentration of the cytoplasm, in turn, triggered the release of the miR146a so that the miRNA could react with RNA interference machinery to effectuate gene silencing. The polyplex was found to inhibit AIPC growth with no detectable systemic toxicity (**Figure** **17**).

**Figure 18:**
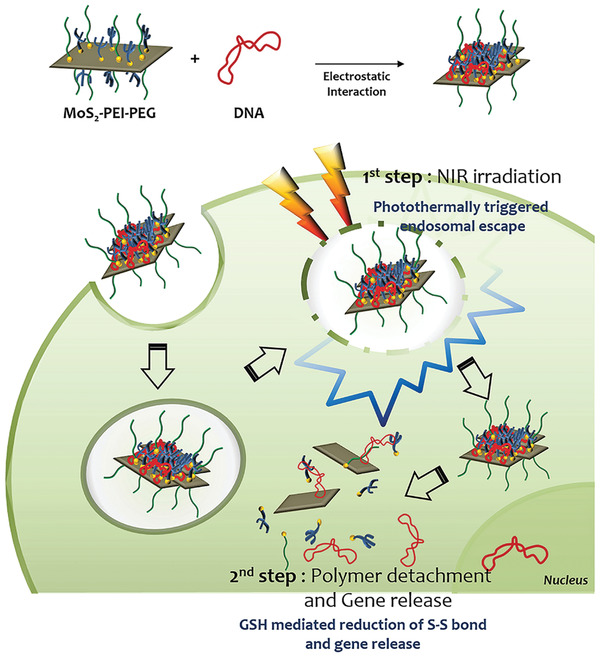
Schematic of plasmid DNA delivery to target tumorous cells using a light‐ and redox‐responsive MoS_2_–PEI–PEG nanocarrier system for sequential photothermally activated endosomal escape followed by redox‐mediated polymer detachment and DNA release. Abbreviation: GSH, glutathione. Reproduced with permission.^[^ ^219^ ^]^ Copyright 2015, Wiley‐VCH.

Changes in acidity is not the only stimulus that scientists use to pair with other stimuli for multiple stimuli‐responsive gene delivery. The synergistic effect between redox‐response and other stimuli has also attracted their attention. A NIR‐ and redox‐responsive gene carrier system consisting nanoscopical 2D MoS_2_ that was modified by the attachment of PEI and PEG via disulfide bonds has been synthesized. This MoS_2_–PEI–PEG nanocomposite interacted with pDNA by electrostatic interaction and produced a highly stable nanocomplex.^[^ ^219^ ^]^ When uptaken by colon carcinoma cells and murine melanoma cells, the use of NIR light irradiation resulted in photothermally triggered endosomal escape. Subsequent polymer detachment and pDNA release was achieved via intracellular glutathione‐mediated reduction of disulfide bonds. This external and internal stimuli‐initiated sequential process enhanced gene delivery without severe cytotoxicity, and provided a spatiotemporally controllable platform for delivery of genetic materials into target cells (**Figure** **18**). Similarly, NIR light and glutathione have been harnessed for the development of another light‐ and redox‐responsive carrier for delivery of p65‐shRNA for treatment of metastatic cancer.^[^ ^220^ ^]^

Scientists also utilize exogenous stimuli for the construction of multiple stimuli‐responsive gene delivery systems. For example, redox‐ and MMP‐responsive polymeric micelles have been designed for co‐delivery of the endogenous tumor suppressor miRNA‐34a and doxorubicin to tumorous cells. The system relied on the generation of an MMP‐2‐sensitive doxorubicin conjugate which responded to increased extracellular concentration of MMP‐2 in a tumorous microenvironment for release of the chemotherapeutic agent. In addition, a glutathione‐sensitive miRNA‐34a conjugate was synthesized to harness the increased intracellular concentration of glutathione in the HT1080 fibrosarcoma cells for release of the miRNA.^[^ ^221^ ^]^ The self‐assembled conjugates were coupled with a PEG corona for stability during delivery and a cell‐penetrating peptide for enhanced intracellular delivery. The dual‐responsive system reduced the viability of the fibrosarcoma cells down to 40% and downregulated the expression of Bcl2 and survivin.

**Figure 19:**
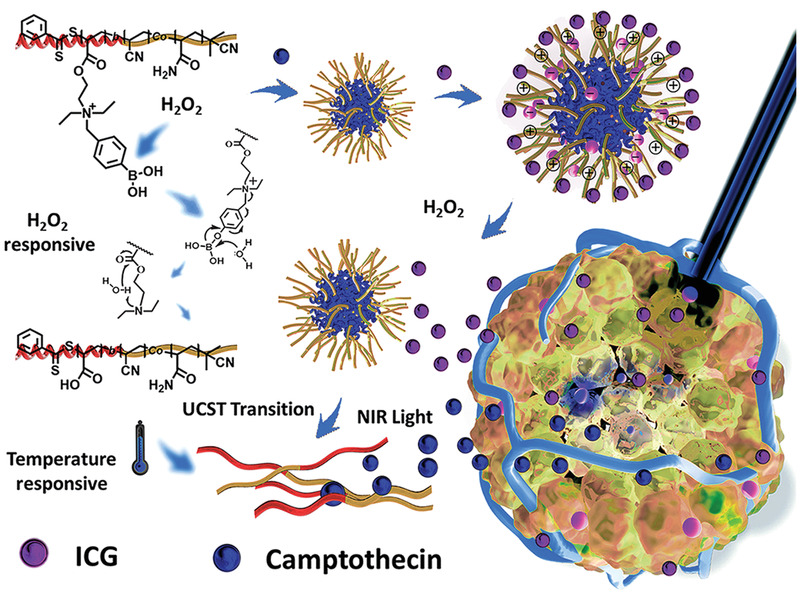
Principle behind the design of a temperature‐ and near‐infrared (NIR) light‐responsive nanoplatform for combined photothermal and chemotherapy against tumor cells. Abbreviations: ICG, idocyanine green; UCST, upper critical solution temperature. Reproduced with permission.^[^ ^223^ ^]^ Copyright 2020, The Royal Society of Chemistry.

In another example of integrated exposure of external and internal stimuli, a functional cargo delivery system with dual responses to temperature and ROS (H_2_O_2_) was designed to target pancreatic cancer cells. The combined chemotherapy and photothermal therapy was designed to achieve a synergistic therapeutic effect.^[^ ^222^ ^]^ Functionalized micelles were constructed from a temperature‐sensitive polymer with an upper critical solution temperature transition segment of poly(acrylamide‐*co*‐acrylonitrile) (poly(AAm‐*co*‐AN)), and a ROS‐responsive block of poly[(2‐acryloyl)ethyl(*p*‐boronic acid benzyl)diethylammonium bromide] (P(B‐DEAEA)). The chemotherapeutic agent camptothecin was encapsulated into the micelles, and the photosensitizer idocyanine green was absorbed onto the micelles via electrostatic inter‐action. The camptothecin and idocyanine green‐loaded micelles effectively accumulated in tumor sites through the “enhanced permeability and retention effect,” a tendency by which liposomes, nanoparticles and macromolecular drugs accumulate in tumor tissue much more than they do in normal tissue.^[^ ^222^ ^]^ In the presence of increased ROS in the tumor microenvironment, the original amino‐based copolymer was converted by e H_2_O_2_ to a carboxy‐based copolymer, releasing the loaded idocyanine green that reduced the size of the micelles to enable them to penetrate the tumorous tissue.^[^ ^223^ ^]^ Upon irradiation by NIR light at 808 nm, the temperature‐responsive micelles underwent upper critical temperature transition, producing heat to kill the tumor cells via photothermal therapy. The generated heat further triggered the transition of the copolymer from a hydrophobic to a hydrophilic state to facilitate release of the loaded camptothecin into the deep tumor cells to achieve a chemotherapeutic effect (**Figure** **19**).

Apart from the use of twin stimuli responses, some studies have also constructed nanocarriers based on triple stimuli responses. A triple‐responsive system that is sensitive to hyaluronidase, ROS and NIR light has already been introduced in Section 4.1.3.^[^ ^137^ ^]^ A triple‐responsive polymer nanoaggregate has been designed that responded to acidic pH, ROS (H_2_O_2_) and UV light (365 nm) based on the unique multicomponent reaction of iminoboronate ester.^[^ ^224^ ^]^ Another triple‐responsive nanocarrier system has been synthesized using hollow porous carbon nanoparticles that were responsive to NIR light irradiation as an external stimulus and glutathione and low intracellular acidity of tumorous cells as the external stimuli.^[^ ^225^ ^]^ Although these triple stimuli‐responsive nanocarrier systems have been demonstrated to effective deliver chemotherapeutic agents such as doxorubicin, their ability to function as nanocarriers for gene delivery have not been demonstrated. However, with the pace in which nonviral vectors for gene delivery is developing, we envisage that systems capable of responding to three or more stimuli will soon be available for treatment of cancer and other genetic diseases that are not amenable to conventional therapy. **Table** **4** represents a brief summary of the mechanisms involved in representative delivery systems that utilize multiple stimuli for cargo release.

**Table 4:** Examples of multiple stimuli for nucleic acid delivery. Additional abbreviations: MMP, matrix metalloproteinase; Ref, reference

Multiple stimuli		Materials	Achieved properties	Refs.
pH	Light	Poly(ethylene glycol)‐*b*‐poly (2‐dimethylaminoethyl methacrylate)‐*b*‐poly(pyrenylmethyl methacrylate) [PEG‐*b*‐PDMAEMA‐*b*‐PPy]	Light‐ and pH‐induced structural transitions of the triblock copolymer micelles to improve the stability and release efficiency of siRNA‐condensed micelleplexes.	^[^ ^217^ ^]^
	Redox	Transgelin‐2 protein [TAGLN2]	Partial degradation of the dual‐responsive nanocomplexes in the acidic milieu of endosomes. Continued degradation in the reducing environment of the cytoplasm to release siRNA.	^[^ ^212^ ^]^
		Methoxypoly(ethylene glycol)‐polylactide ‐polyhistidine‐*ss*‐oligoethylenimine [mPEG‐*b*‐PLA‐PHis‐*ss*OEI]	The polyplex showed good encapsulation of DOX and siRNA as well as triggered payload release in response to pH/redox stimuli because of protonation and disulfide bond cleavage.	^[^ ^214^ ^]^
		Methoxy‐poly(ethylene glycol)‐*b*‐polylactide ‐polyhistidine‐*ss*‐polyethylenimine [PEG‐*b*‐PLA‐PHis‐*ss*PEI]	siRNA disassembly was triggered by sensing the acidity and redox potential in endosomes. Efficient endosomal escape of siRNA was facilitated via endosomal membrane destabilization of detached PEI as well as the “proton sponge effects.”	^[^ ^213^ ^]^
	Magnetic field	T‐PBP = Vitamin A decorated–polyethylene glycol–polyethyleneimine–poly(*N*‐(*N*′,*N*′‐diisopropylaminoethyl)‐cobenzylamino) aspartamide [VA–PEG–bPEI–PAsp(DIP–BzA] T‐PBP@superparamagnetic iron oxide [T‐PBP@SPIO]	pH‐sensitive T‐PBP micelles efficiently transported miRNA‐29b and miRNA‐122 to hepatic stellate cells in a magnetic resonance imaging‐visible manner. This resulted in a synergistic antifibrosis effect via downregulating the expression of fibrosis‐related genes.	^[^ ^216^ ^]^
	ATP	Polyethylenimine‐(4‐(bromomethyl)phenylboronic acid)/miR146a/polyethylenimine‐(2,3‐dimethylmaleic anhydride)‐cetuximab [PEI−PBA/miR146a/PEI−DMA‐C225]	pH/ATP‐activated nanocomplexes for increasing cytosolic delivery of miR146a. Effectively inhibited the expression of epidermal growth factor receptor in androgen‐independent prostate cancer.	^[^ ^218^ ^]^
	Temperature	Poly(ethylene oxide)‐block‐poly (*N*‐isopropylacrylamide ‐stat‐7‐(2‐ methacryloyloxyethoxy)‐4‐methylcoumarin‐stat‐2‐(diethylamino)ethyl methacrylate. [PEO‐*b*‐P(NIPAM‐stat‐CMA‐stat‐DEA)]	Temperature‐ and pH‐sensitive P(NIPAM‐stat‐CMA‐stat‐DEA) block Formed dual‐gated heterogeneous membranes. The temperature‐controlled “boarding gate” could be opened at room temperature for facile encapsulation of siRNA and pDNA into the polymersomes directly in aqueous solution.	^[^ ^211^ ^]^
		*N*‐isopropylacrylamide [IPAAm]	Both micelle variations demonstrated enhanced intracellular uptake under mildly acidic (pH 6.8) conditions at temperatures slightly above their lower critical solution temperatures. Minimal uptake at physiological (pH 7.4) conditions.	^[^ ^210^ ^]^
Redox	MMP‐2	1,2‐Distearoylsn‐glycero‐3‐phosphoethanolamine‐*N*‐(methoxy(polyethylene glycol)‐ 1000) [PEG1k‐PE]	The MMP‐2 sensitive system resulted in threefold high cytotoxicity in MMP2‐overexpressing HT1080 cells. Inhibited proliferation and migration of HT1080 cells. Sensitivity to glutathione reduced cell viability and resulted in cell death.	^[^ ^221^ ^]^
	Light	RGD–PEG–DSPEI–GNR/short hairpin RNA [RDG/shRNA]	Dual‐stimulation from near‐infrared laser irradiation and high intracellular glutathione concentrations inhibited cancer cell proliferation and invasion.	^[^ ^220^ ^]^
		A 2D nanomaterial, MoS_2_, was modified by attaching two polymers (polyethylenimine and polyethylenglycol) via disulfide bonds [MoS_2_–PEI–PEG]	Photothermal conversion of MoS_2_ nanosheet was used to induce photothermally triggered endosomal escape upon irradiation with near infrared light. After endosomal escape, polymers detached from the MoS_2_ nanosheet by glutathione, resulting in effective gene release from the nanocomposite.	^[^ ^219^ ^]^
	MMP‐9	Hyperbranched poly(amido amine)‐methotrexate [HPAA‐MTX]	Disulfide bonds were cleaved by glutathione. HPAA‐MTX possessed excellent degradation property in response to a reducing intracellular environment. This phenomenon was attributed to the abundant disulfide bonds present in the HPAA segment.	^[^ ^226^ ^]^
	Hyaluronic acid	Upconversion nano‐onions: upconversion nanoparticles‐rose bengal conjugated polyethylenimine‐diselenide bonds connected polyethylenimine‐cell‐penetrating peptide R8‐hyauronic acid [UCNPs‐PEIRB‐PEISeSe/siRNA‐R8‐HA]	Hyaluronic acid prevented siRNA leakage during delivery and specifically targeted tumor cells with overexpressed CD44 membrane receptors. Hyaluronic acid digested by cells that secrete hyaluronidase. The intracellular signaling reactive oxygen species triggered intracellular disassembly of the nanocarriers with corresponding drug release.	^[^ ^137^ ^]^
		Hyaluronic acid/hyaluronidase/chitosan/short hairpin RNA [Hyal/HAase/CS/l/shRNA]	The acid protease hyaluronidase is negatively charged at pH 7.0. Increased drug penetration into the malignancy by decreasing intratumoral pressure and loosening the intercellular connective matrix.	^[^ ^215^ ^]^
		Disulfide crosslinked hyaluronic acid [HA‐SS‐HA]	Redox‐responsive delivery systems with crosslinked disulfide bonds maintain adequate stability in the circulation and extracellular milieu before undergoing rapid cleavage when exposed to the reducing conditions within the cell.	^[^ ^121^ ^]^
Ultrasound and light		Polyethylenimine‐DNA complex/NIR797 fluorescent dye/gold microbubbles [PEI‐DNA/NIR797/AuMBs]	Near infrared light application enables precise determination of tumor position. Ultrasound‐targeted, site‐specific payload delivery.	^[^ ^227^ ^]^
Light, acid and H_2_O_2_		Poly(ethylene glycol)‐iminoboronate nitrobenzyl ethanediol chelate [PEG‐INEC]	Stimulation of the multiple triggers results in synergistic release of encapsulated hydrophobic molecules in water. Practical applications in drug and gene delivery.	^[^ ^224^ ^]^

Because the human body is a complex environment, the sensitivity of a single response mode is often insufficient because of the insignificant difference between internal microenvironmental factors. For this reason, multiresponse systems have been designed to further improve the responsiveness of the vector, increase its targeting to the lesion site and reduce toxicity and side effects in in vivo therapy. These multiresponse systems take advantage of the synergistic effect between different responsiveness to achieve a highly sensitive response. At the same time, intelligent regulation may be carried out during the process of gene delivery to overcome obstacles such as poor gene loading capacity, weak endosome/lysosome escape ability, slow gene release, high vector toxicity, and difficult nuclear transport, with the ultimate goal of improving the efficacy of gene transfection.^[^ ^228^, ^232^ ^]^ However, the stability of multiple stimuli‐responsive nanocarriers is poor, the preparation process and therapeutic mechanism are complex and the toxicity of the carrier is relatively high. Moreover, deletion of one stimulus may lead to the failure of the entire gene delivery system.

## 5 Concluding Remarks and Future Perspectives

Recent advances in the comprehension of the molecular mechanisms involved in human diseases have enormously expanded the global demand for gene therapy. Diseases in which gene therapy is utilized for treatment may be categorized into cancers, neurological diseases, rare genetic diseases, cardiovascular disorders and infectious diseases. Among these diseases, cancers and monogenic diseases have commanded the largest share of the demand for gene therapy. Vector technology has been the major obstacle to the clinical application of this paradigm‐shifting treatment modality. Viral vectors have high transfection efficacy inherited from millions of years of evolution. However, they possess inherent shortcomings such as limited genetic material packaging capacity, complex production schemes, broad tropism, cytotoxicity, immunogenicity and tumorigenicity. Nonviral vectors have the potential to address many of the aforementioned issues. Nevertheless, research and application of nonviral nanocarriers are still in their infancy. In particular, most of the nanoscopical nonviral vectors reviewed here have not undergone clinical testing; 90% of the clinical trials conducted to date are associated with the use of viral vectors.

Adoption of gene therapy as a routine clinical procedure requires extensive research on toxicity and safety, as well as in the design of cost‐effective and facile vector synthesis protocols. To improve the efficacy of nonviral gene delivery to the level of viral vectors, it is necessary to understand the challenges faced by nonviral gene delivery vectors in vitro and in vivo. The present review covers recent research on stimulus‐responsive nanocarriers in gene therapy, summarizing the rapid developments over the last five years that lay the foundation for the design of more effective nonviral vectors for treatment of genetic diseases. Intracellular delivery is a major challenge for nonviral gene delivery vectors because of multiple barriers to transport pathways, such as endocytosis, endosomal/lysosomal escape and intracellular release. Initial efforts on the development of nonviral vectors focused on decorating the surface of nanomaterials with hydrophilic nonionic polymer chains (such as PEG, PEI or polysaccharide) to reduce phagocytosis, absorption and removal of nanomaterials from the blood stream by macrophages. By reducing the removal rate, the circulation half‐life of nanocarriers increases, which provides additional opportunity for the nanocarriers to traverse blood vessel walls.

There are challenges associated with the clinical translation of stimulus‐responsive gene delivery systems. First, the transfection efficiency of nonviral nanocarriers is still low. Second, although PEGylated nanocarriers have shown advantages in extending circulation, further improvements are needed to avoid premature release of the nanocarriers in the blood circulation.^[^ ^229^ ^]^ Third, the complex human microenvironment may make it difficult for genes to be released completely in targeted cells without severe off‐target effects. Finally, safety remains an issue that must be considered. Although the immunogenicity of stimulus‐responsive gene delivery systems is relatively low, the maximum‐tolerated dosage and long‐term toxicity of nanoparticles should be fully evaluated because of their physicochemical properties.^[^ ^230^ ^]^

With the interdisciplinary development of materials science, molecular biology, oncology, pharmacy and other disciplines, researchers will undoubtedly overcome the hurdles associated with clinical translation of stimulation‐responsive nanocarriers. Future breakthroughs may include the following aspects. First, better appreciation of the obstacles faced by nonviral gene delivery vectors in vitro and in vivo, and develop other efficient delivery routes apart from the EPR effect. Second, the development of current delivery systems relies excessively on animal experiments (e.g., mice). However, the fundamental physiological differences between humans and mice can easily lead to failure in clinical translation. In the future, with better understanding of the unique biological signals of diseases such as tumors, precise release of therapeutic nucleic acids at target sites can be achieved. Finally, gene delivery is a multistep process; future research have to focus on the structural design and synthesis of multiple stimuli‐responsive nanocarriers that can overcome various obstacles in gene delivery while improving their biosecurity.

**Figure 20:**
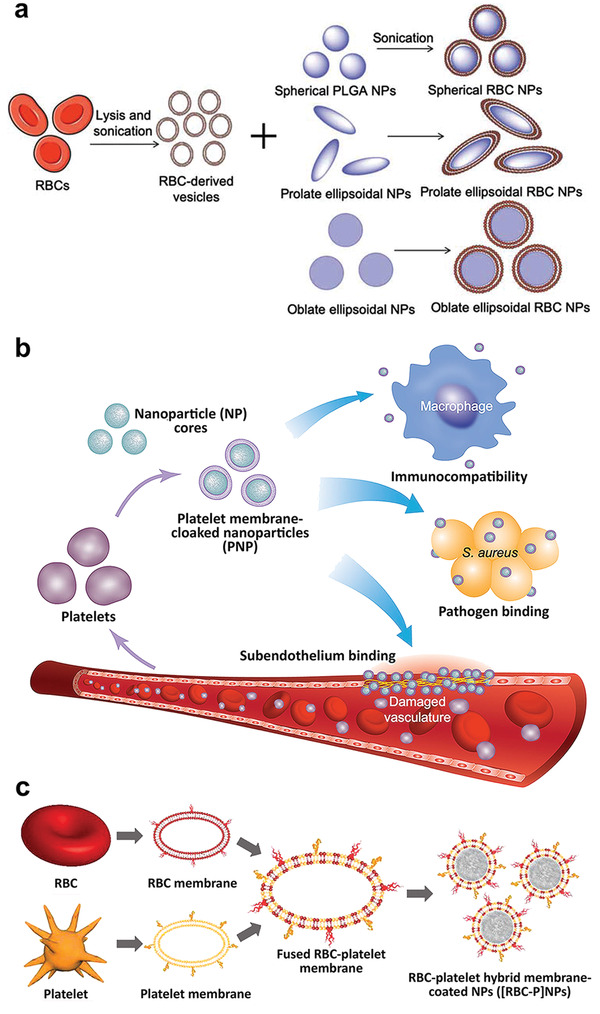
Development of biological membrane‐coated nanoparticles as potential biocompatible gene delivery vectors. a) Schematic of the preparation of red blood cell (RBC) membrane‐coated poly(lactic‐*co*‐glycolic) acid (PLGA) nanoparticles (NP). b) PLGA nanoparticles enclosed entirely in plasma membrane derived from human platelets. The resulting nanoparticles possess platelet‐mimicking properties. c) Fabrication of red blood cell‐platelet hybrid membrane‐coated PLGA nanoparticles. The hybrid membrane is derived from both RBCs and platelets and then fused together. a) Reproduced with permission.^[^ ^232^ ^]^ Copyright 2020, American Association for the Advancement of Science. b) Reproduced with permission.^[^ ^233^ ^]^ Copyright 2015, Springer Nature. c) Reproduced with permission.^[^ ^234^ ^]^ Copyright 2017, Wiley‐VCH.

Passive accumulation in nonspecific body tissues may be reduced by active targeting of pathological tissues, via the attachment of appropriate antibodies, peptides or small molecular ligands attached to the nanomaterials. To date, most transfection agents and nanoparticle carriers used for intracellular nucleic acid delivery cannot differentiate between pathological cells and healthy cells, which may result in adverse health consequences. A colossal challenge faced with the clinical translation of gene therapy is the development of novel gene carriers that can efficiently transfect with low toxicity and only silence the target protein in pathological tissues. One of the bottlenecks in nanomedicine is the biological compatibility of nanomaterial‐based drug and gene carriers, which impose severe restrictions on their therapeutic efficacy. Nonviral vectors must be able to tolerate the challenges of a physiological environment in order for them to be clinically relevant.^[^ ^231^ ^]^ In an early study published in 2011, polymeric nanoparticles were functionalized (camouflaged) with plasma membranes derived from erythrocytes to enhance cargo delivery by increasing their circulation time via evasion of macrophage uptake (**Figure** **20**a).^[^ ^232^ ^]^ Utilizing a similar “artificial cell” principle, a recent work reported that nanoparticles coated with the plasma membrane derived from human blood platelets are much less likely to be consumed by macrophages, compared with uncoated nanoparticles.^[^ ^233^ ^]^ The resulting particles possess platelet‐mimicking properties in terms of immunocompatibility and subendothelium binding, and did not induce complement activation in the body. Moreover, the coated nanoparticles can be selectively adsorbed by platelet‐related disease substances for specific targeted therapy (Figure 20b). A more recently published work expanded this “artificial cell” concept further by taking advantage of the natural properties of plasma membranes derived from erythrocytes and platelets, producing biomimetic fused membranes‐functionalized nanoparticles (Figure 20c).^[^ ^234^ ^]^ Although artificial cell‐like biomimetic nanocarrier systems have not been adopted for gene delivery, the principle behind their fabrication should provide a candid roadmap for future development in this important facet of genetic nanomedicine.

The advent of contemporary gene‐editing technologies such as CRISPR, transcription activator‐like effector nucleases and zinc finger nucleases have enabled scientists to perform precise genomic modifications at destined human genome locations. The last five years have seen commencement of clinical trials based on genome‐editing technologies, particularly for the CRISPR/Cas systems.^[^ ^3^ ^]^ Multiple viral and nonviral delivery approaches have been explored for in vivo delivery of CRISPR components for genome‐editing. Because of the large size of CRISPR nucleases, loading of all the genome‐editing components into a single vector presents a huge challenge in therapeutic applications. For example, the size limit of gene insertion of the AAV viral vector is around 4700 base pairs. In comparison, *Streptococcus pyogenes* Cas9 alone comprises ≈4300 base pairs, which renders incorporation of additional CRISP components such as sgRNA ribonucleoprotein, pDNA or reporter gene close to impossible for a single viral vector.^[^ ^235^ ^]^ Ongoing pursuits for more efficient nonviral delivery vehicles have utilized lipid‐based delivery vehicles based on cationic lipids such as 1,2‐dioleoyl‐3‐trimethylammonium‐propane (DOTAP) or 1,2‐di‐*O*‐octadecenyl‐3‐trimethylammonium propane (DOTMA). Although these vehicles provide better cargo loading capacity and acceptable endosomal/lysosomal release, these formulations suffer from cytotoxicity issues because of their consistent positive charges. To circumvent these challenges, lipid nanoparticles based on ionizable cationic lipids have become available recently.^[^ ^236^ ^]^ Currently available lipid nanoparticles are more adept at delivering siRNA. They are not well suited for larger payloads such as CRISPR/Cas systems. Accordingly, novel lipid nanoparticles that have the capacity for loading longer nucleic acids have to be developed for transfection of CRISPR/Cas systems. Future research may exploit the use of inorganic nanoparticles such as gold nanoparticles for the delivery of genome‐editing components.^[^ ^237^ ^]^

Although innovations in carrier technology and safety issues are paramount for the clinical translation of gene therapy medicinal products, acceptance of these strategies by the population at large ultimately relies on how gene therapy regimes are regulated. While gene therapy provides unprecedented hopes for complete cure of rare genetic diseases and cancers, they also raise earnest fears regarding the often intangible and not‐yet‐foreseeable risks that accompany these irreversible forms of one‐time treatments. For real, the general public has been haunted by deaths of the recruited subjects both in the US and in Europe, albeit small in number compared with deaths caused by pandemics such as the Spanish flu or COVID‐19. The responsibility for balancing aspirations and fears in the use of gene delivery systems lie within government agencies. In the United States, the Department of Health and Human Services (DHHS) is the gatekeeper that is charged with overseeing the running of clinical trials. Two organizations within the DHHS, the Office for Human Research Protections and the FDA, are authorized to ensure that all investigators comply with the associated regulations when conducting clinical gene therapy trials. In Europe, the European Medicines Agency issues scientific guidelines on gene therapy to help developers prepare marketing authorization applications. The Committee for Advanced Therapies is responsible for making marketing authorization recommendations to the Committee of Human Medicinal Products. In China, the Ministry of Health is the regulatory health authority responsible for developing laws, regulations and policies for gene therapies conducted in China. In 2009, the Chinese Ministry of Health classified cellular therapies as category three medical technologies. This category of medical technologies is considered to have the highest risk, posing the most challenging ethical issues that have not yet been scientifically proven for their therapeutic values. As such, approval from a technical audit board is required prior to the use of a gene therapy product. Nevertheless, China has not yet formed a comprehensive and systematic legal regulatory system for human genetic technology issues. There has been no direct regulation on gene therapy at the legal level prior to the emergence of the CRISPR baby incident.^[^ ^2^ ^]^

The statement published in by the US National Institutes of Health in August 2018 that gene therapies no longer require review prior to the commencement of clinical studies appeared to have injected confidence to the lay public regarding the long‐term safety of gene therapy regimes: “There is no longer sufficient evidence to claim that the risks of gene therapy are entirely unique and unpredictable—or that the field still requires special oversight that falls outside our existing framework for ensuring safety.”^[^ ^238^ ^]^ Nevertheless, there are still opinions around that long‐term safety issues have not been evaluated completely to emphatically declare that today's gene therapies are safe. There are still unanswered questions, such as the possibility of autoimmune‐like scenarios in which the body's immune system fail to recognize the new proteins created by gene therapies as “self,” as well as the legal issues and consequences associated with nonregulated use of emerging gene‐editing therapies. Gene therapy also requires follow‐up for patients that extends for years after product approval because the long‐term effects of these one‐time treatments are simply unknown, such as the possibility of inducing tumor‐suppressor genes that lead to the development of tumors. While there have been claims that “overregulation is an unnecessary hindrance to human gene therapy,”^[^ ^239^ ^]^ the CRISPR‐baby incident has re‐ignited the debate on whether existing legal mechanisms are adequate in regulating clinical studies on human gene therapy. This is a candid issue for readers to ponder.

## Conflict of Interest

The authors declare no conflict of interest.
